# Synthesis, Pharmacological Profile and Docking Studies of New Sulfonamides Designed as Phosphodiesterase-4 Inhibitors

**DOI:** 10.1371/journal.pone.0162895

**Published:** 2016-10-03

**Authors:** Isabelle Karine da Costa Nunes, Everton Tenório de Souza, Suzana Vanessa S. Cardozo, Vinicius de Frias Carvalho, Nelilma Correia Romeiro, Patrícia Machado Rodrigues e Silva, Marco Aurélio Martins, Eliezer J. Barreiro, Lídia Moreira Lima

**Affiliations:** 1 Instituto Nacional de Ciência e Tecnologia de Fármacos e Medicamentos (INCT-INOFAR). Laboratório de Avaliação e Síntese de Substâncias Bioativas (LASSBio®), Instituto de Ciências Biomédicas, Universidade Federal do Rio de Janeiro, Rio de Janeiro, Brasil; 2 Programa de Pós-graduação em Farmacologia e Química Medicinal, Instituto de Ciências Biomédicas, Universidade Federal do Rio de Janeiro, Rio de Janeiro, Brasil; 3 Laborat×rio de Inflamação, Instituto Oswaldo Cruz-Fiocruz, Rio de Janeiro, Brasil; Centre National de la Recherche Scientifique, FRANCE

## Abstract

Prior investigations showed that increased levels of cyclic AMP down-regulate lung inflammatory changes, stimulating the interest in phosphodiesterase (PDE)4 as therapeutic target. Here, we described the synthesis, pharmacological profile and docking properties of a novel sulfonamide series (**5** and **6a-k**) designed as PDE4 inhibitors. Compounds were screened for their selectivity against the four isoforms of human PDE4 using an IMAP fluorescence polarized protocol. The effect on allergen- or LPS-induced lung inflammation and airway hyper-reactivity (AHR) was studied in A/J mice, while the xylazine/ketamine-induced anesthesia test was employed as a behavioral correlate of emesis in rodents. As compared to rolipram, the most promising screened compound, **6a** (LASSBio-448) presented a better inhibitory index concerning PDE4D/PDE4A or PDE4D/PDE4B. Accordingly, docking analyses of the putative interactions of LASSBio-448 revealed similar poses in the active site of PDE4A and PDE4C, but slight unlike orientations in PDE4B and PDE4D. LASSBio-448 (100 mg/kg, oral), 1 h before provocation, inhibited allergen-induced eosinophil accumulation in BAL fluid and lung tissue samples. Under an interventional approach, LASSBio-448 reversed ongoing lung eosinophilic infiltration, mucus exacerbation, peribronchiolar fibrosis and AHR by allergen provocation, in a mechanism clearly associated with blockade of pro-inflammatory mediators such as IL-4, IL-5, IL-13 and eotaxin-2. LASSBio-448 (2.5 and 10 mg/kg) also prevented inflammation and AHR induced by LPS. Finally, the sulfonamide derivative was shown to be less pro-emetic than rolipram and cilomilast in the assay employed. These findings suggest that LASSBio-448 is a new PDE4 inhibitor with marked potential to prevent and reverse pivotal pathological features of diseases characterized by lung inflammation, such as asthma.

## Introduction

Asthma is a chronic lung disease characterized by bronchoconstriction and inflammation of the airways. According to WHO, 235 million people suffer from asthma and over 80% of asthma deaths occurs in low and lower-middle income countries [1]. Its therapy includes inhaled β-agonists, corticosteroids, and mast cell stabilizers, and systemic medications as exemplified by leukotriene receptor antagonists, oral corticosteroids and, more recently, by biological drugs such as anti-IgE, anti-IL-5 and anti-IL-13 [2].

**Fig 1:**
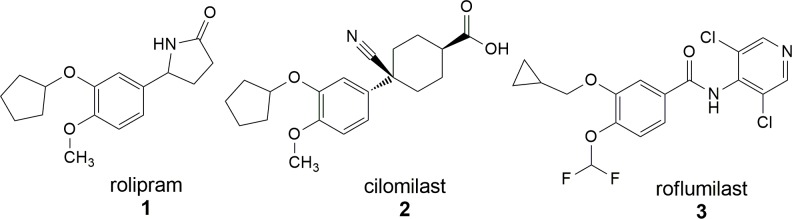
Examples of PDE4 inhibitors of first and second generations.

Several inflammatory cells are involved in asthma pathogenesis, being activated T cells and eosinophils important pathophysiological features in this pulmonary disease, while mast cells activation has a central role in the initial response to allergen in sensitized individuals. Ongoing mast cell degranulation, several mediators are delivered, such as histamine, prostaglandin D_2_, leukotriene C_4_, tryptase and pro-inflammatory cytokines [3]. The assumption that biological response triggers in asthma can be modulated by the levels of cyclic nucleotides aroused the interest in phosphodiesterases (PDE), as targets for new drugs to treat asthma and others pulmonary diseases. These enzymes are involved in many signaling processes and hydrolyze two of the most important signaling molecules in cells, cAMP and cGMP. To date, 21 PDE isoforms were recognized and grouped into 11 families (PDE1-PDE11) [4]. PDE4, that specifically hydrolyses cAMP, is encoded by 4 distinct genes (PDE4A, PDE4B, PDE4C, PDE4D) and is predominant in inflammatory cells including mast cells, eosinophils, neutrophils, T cells and so on. It plays an important role in inflammatory and immunomodulatory responses [5, 6]. Several PDE4 inhibitors were developed and their effectivity in asthma models were stablished. Since the identification of roplipram (1), the first generation of PDE4 inhibitor, until the discovery of cilomilast (2) and roflumilast (3), both approved to the treatment of inflammatory airway diseases (Fig 1) [7, 8], the challenge in the development of new PDE4 inhibitors is based on the ability of circumvent the main side-effect of this therapeutic class, represented by its capability to induce emesis [9].

In this paper we described the synthesis, pharmacological profile and docking study of new sulfonamides (**5** and **6a-k**) designed as PDE4 inhibitors.

**Fig 2:**
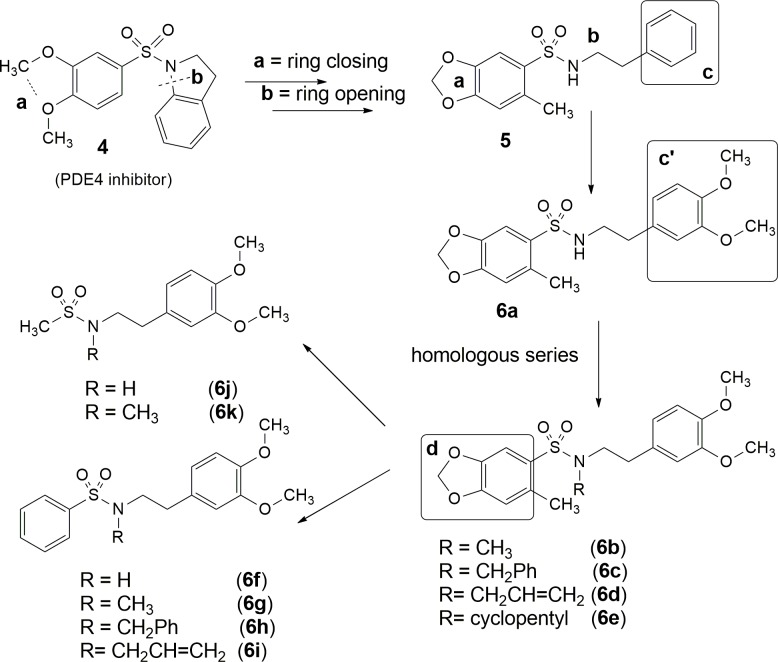
Genesis concept of sulfonamides (5 and 6a-k) designed as PDE4 inhibitors.

The sulfonamide **5** was designed by molecular modification on the structure of prototype **4**, previously described by Montanna and coworkers as a PDE-4 inhibitor [10]. The modifications were based on non-classical bioisosterism, represented by ring closing (**a**, Fig 2) and ring opening (**b**, Fig 2) [11]. A congener series was designed from compound **5** in order to introduce the dimethoxy substituent in ring **c**, originating the 3,4-dimethoxy phenyl subunit (**c’**), considered an important pharmacophore to PDE 4 recognition. Later, a homologous series (**6a-e**) was designed and a molecular simplification was proposed, based on the replacement of 1,3-benzodioxole moiety (**d**) by a phenyl (**6f-i**) and methyl groups (**6j-k**) (Fig 2).

## Materials and Methods

### Synthesis and Characterization of Compounds

#### Chemical

In this study we have reported the synthesis of new sulfonamides derivatives. Reagents and solvents were purchased from commercial suppliers and used as received. The progress of all reactions was monitored by thin layer chromatography (TLC), which was performed on 2.5 × 7.5 cm^2^ aluminum sheets precoated with silica gel 60 (HF-254, E. Merck) to a thickness of 0.25 mm. The developed chromatograms were viewed under ultraviolet light (254 nm). IR spectra (cm^−1^) were taken on FTLA spectrometer in KBr discs. Analytical HPLC was used for compound purity determinations using Shimadzu LC-20AD with or Kromasil 100-5C18 (4.6 mm × 250 mm) and a Shimadzu SPD-M20A detector at 254 nm wavelength. The solvent system used for HPLC analyses was acetonitrile: water (70:30 and 60:40), with or without of 0.1% trifluoroacetic acid. The isocratic HPLC mode was used, and the flow rate was 1.0 mL/min. The purity of compounds was found to be greater than 95%. ^1^H e ^13^C NMR were determined using a Bruker AC 200 spectrometer, 500, 200, 125 e 50 MHz, respectively, in CDCl_3_ deuterated containing *ca*. 1% tetramethylsilane as an internal standard. The peak positions are given in parts per million (δ ppm), and J values are given in hertz. Signal multiplicities are represented by: s (singlet), d (doublet), t (triplet), q (quadruplet), m (multiplet) and br (broad signal). Melting points were determined with a Quimis 340 apparatus and are uncorrected. The HPLC solvents (methanol, acetonitrile and dimethylsulfoxide) were purchased from Sigma-Aldrich (St. Louis, MO, USA). Water used in the preparations has been previously purified and filtered using a Milli-Q system (Millipore, St Quentin-en-Yvelines, France).

#### Procedure to prepare 2-methyl-3,4-methylenedioxybenzenesulfonyl chloride (9)

The key intermediate 2-methyl-3,4-methylenedioxybenzenesulfonyl chloride (**9**) was obtained in accordance to the methodology described by Lima et al. (1999) [36].

#### General procedure for the preparation of Sulfonamides

Sulfonamides **5** and **6a-k** were prepared by a condensation reaction of the corresponding chloride derivative (0.5g) which was solubilized in dichloromethane (15 mL), containing 50 μL of triethylamine, with respective functionalized amines: 2-phenylethylamine and 3,4-dimethoxyphenethylamine (1 mmol) to obtain **5** and **6a-k,** respectively. The mixture was stirred at room temperature for 2-3h. Afterward, the isolation was carried out with dilution using 10mL of dichloromethane and extracted with aqueous HCl 10% (four times with 10 ml each time). The organic phase was dried with addition of anhydrous sodium sulfate, filtered and concentrated under reduced pressure to obtain the compounds. Yields and characterization pattern are described below:

#### 6-methyl-*N*-phenethylbenzo[d][1,3]dioxole-5-sulfonamide (5) LASSBio-964

The title compound was obtained in 81% yield, by condensing **9** with 2-phenylethylamine, as a white powder with mp 88–89°C.

**IR (KBr) (cm**^**-1**^**):** 3243 (ν NH), 1251,1043 (ν CO), 1453 (ν CH_2_), 1432 (ν CH_3_);

^**1**^**H NMR (200 MHz, CDCl**_**3**_**) *δ* (ppm):** 2.37 (s, 3H, Ar-CH_3_), 2.78 (t, 2H, -CH_2_-Ar, *J* = 8 Hz), 3.20 (q, 2H, N-CH_2_-CH_2,_ *J* = 8 Hz e *J* = 6 Hz), 4.57 (t, 1H, NH, *J* = 6 Hz), 6.03 (s, 2H, O- CH_2_ -O), 6.70 (s, 1H, H_5_), 7.09–7.30 (m, 5H, Ph), 7.45 (s, 1H, H2’);

^**13**^**C NMR (50 MHz, CDCl**_**3**_**) *δ* (ppm):** 19.9 (Ar-CH_3_), 35.6 (CH_2_-Ar), 43.9 (N-CH_2_-), 101.9 (O-CH_2_-O), 109.9 (Ar-C_2´_), 112.0 (Ar-C_5’_), 126.7 (Ar-C_4_), 112.1 (Ar-C_5_), 128.6 (Ar-C_5,_ C_3_), 128.7 (Ar-C_6,_ C_2_), 130.3 (Ar-C_6´_), 132.8 (C_1’_), 137.6 (Ar-C_1_), 145.6 (Ar-C_3’_), 150.9 (Ar-C_4’_).

**HPLC**: 60/40 acetonitrile/water; **λ** 254 nm: 97,2% purity

#### *N*-(3,4-dimethoxyphenethyl)-6-methylbenzo[d][1,3]dioxole-5-sulfonamide (6a) LASSBio-448

The title compound was obtained in 70% yield, by condensing **9** with 2,3-dimethoxyphenylethylamine, as a powder with mp 145−146°C.

**IR (KBr) (cm**^**-1**^**):** 3305 (ν NH), 2922, 1450 (ν CH_2_), 2967, 1421 (ν CH_3_), 1259,1028 (ν CO);

^**1**^**H NMR (200 MHz, CDCl**_**3**_**) *δ* (ppm):** 2.36 (s, 3H, Ar- CH_3_), 2.72 (t, 2H, CH_2-_CH_2_-Ar, *J* = 8 Hz) 3.16 (q, 2H, N-CH_2_-CH_2,_ *J* = 8 Hz e *J* = 6 Hz), 3.82 (s, 3H, O-CH_3_), 3.85 (s, 3H, O-CH_3_), 4.63 (t, 1H, NH, *J* = 6 Hz), 6.02, (s, 2H, O-CH_2_-O), 6.60–6.78 (m, 4H, H_2_, H_5_, H_6_, H_5’_), 7.41 (s, 1H, H_2’_);

^**13**^**C NMR (50 MHz, CDCl**_**3**_**) *δ* (ppm):** 20.1 (CH_3_-Ar), 35.3 (CH_2_-Ar), 44.1 (N-CH_2_-), 55.9 (O- CH_3_), 56.0 (O- CH_3_), 102.1 (O- CH_2_ -O), 110.9 (Ar-C_2’_), 111.5 (Ar-C_5_), 111.8 (Ar-C_2_), 112.0 (Ar-C_5´_), 120.8 (Ar-C_6_), 130.3 (Ar-C_6’_), 130.5 (Ar-C_1_), 132.9 (Ar-C_1’_), 145.7 (Ar-C_3’_), 148.0 (Ar-C_4_), 149.2 (Ar-C_3_), 151.0 (Ar-C_4’_);

**HPLC:** 60/40 acetonitrile/water; **λ** 254 nm: 98% purity.

#### *N*-(3,4-dimethoxyphenethyl)benzenesulfonamide (6f) LASSBio-965

The title compound was obtained in 53% yield, by condensing benzenesulfonyl chloride with 3,4-dimethoxyphenylethylamine, as a white powder with mp 86–88°C.

^**1**^**H NMR (500 MHz, CDCl**_**3**_**) *δ* (ppm):** 2.70 (t, 2H, CH_2_-CH_2_-Ar, *J* = 6,5 Hz), 3.18 (t, 2H, N-CH_2_-CH_2,_ *J* = 6 Hz), 3.77 (s, 3H, O- CH_3_), 3.81 (s, 3H, O- CH_3_), 5.04 (s, 1H, NH), 6.58 (s, 1H, H_2_), 6.61 (d, 1H, H_6_, *J* = 8,5 Hz), 6.72 (d, 1H, H_5,_ *J* = 8,5 Hz), 7.46 (t, 2H, H_3´_, H_5´,_ *J* = 7 Hz), 7.55 (t, 1H, H_4´,_ *J* = 7 Hz), 7.79 (d, 2H, H_2´_, H_6´_, *J* = 7,5 Hz).

^**13**^**C NMR (125 MHz, CDCl**_**3**_**) *δ* (ppm):** 35.3 (-CH_2_-Ar), 44.4 (N-CH_2_-), 55.8 (2C, O- CH_3_), 111.4 (Ar-C_2_), 111.8 (Ar-C_5_), 120.7 (Ar-C_6_), 126.9 (Ar-C_2´_, Ar-C_6´_), 129.0 (Ar-C_3´_, Ar-C_5´_), 130.2 (Ar-C_4´_), 132.5 (Ar-C_1_), 139.8 (Ar-C_1´_), 147.7 (Ar-C_4_), 149.0 (Ar-C_3_).

**HPLC:** 60/40 acetonitrile/water; λ 254nm: 97,6% purity.

#### *N*-(3,4-dimethoxyphenethyl)methanesulfonamide (6j) LASSBio-985

The title compound was obtained in 83% yield, by condensing methanesulfonyl chloride with 2,3-dimethoxyphenylethylamine, as a powder with mp 73–74°C.

**IR (KBr) cm**^**-1**^: 3241, 1593 (N-H), 1457, 1349 (CH_3_), 1321, 1156 (S = O), 1264, 1024 (C-O).

^**1**^**H NMR (500 MHz, CDCl**_**3**_**) *δ* (ppm):** 2.82 (t, 2H, CH_2_-Ar, *J* = 7 Hz), 2.85 (s, 3H, S-CH_3_), 3.37 (q, 2H, NH-CH_2_-, *J* = 6 Hz, *J* = 7 Hz), 3.86 (s, 3H, O-CH_3_), 3.88 (s, 3H, O-CH_3_), 4.51 (t, 1H, NH, *J* = 6 Hz), 6.73–6.76 (m, 2H, H_2,_ H_6_), 6.82 (d, 1H, H_5,_ *J* = 8,5 Hz);

^**13**^**C NMR (125 MHz, CDCl**_**3**_**) *δ* (ppm):** 36.0 (-CH_2_-Ar), 40.2 (S-CH_3_), 44.5 (N-CH_2_-), 55.9 (2C, O-CH_3_), 111.4 (Ar-C_2_), 111.9 (Ar-C_5_), 120.8 (Ar-C_6_), 130.2 (Ar-C_1_), 147.9 (Ar-C_4_), 149.1 (Ar-C_3_);

**HPLC:** 60/40 acetonitrile/water; λ 254 nm: 97,7% purity.

#### General procedure for the preparation of *N*-alkyl Sulfonamides[12]

The selective *N*-alkylation of compounds **6a**, **6f** and **6j** (0.2g) was performed in the presence of K_2_CO_3_ (1.91 mmol) and acetone (5mL) as solvent. The suspension was thoroughly mixed under vigorous stirring for 5 min, and then the appropriate alkyl reagent was added (1.91 mmol). The reaction was heated at 40°C and maintained under stirring for 5–48 h under reflux. Afterward, the reaction was evaporated under reduced pressure; the residual solid was suspended in 2 mL of AcOEt and then poured into cold water. Purification was achieved by simple filtration, washing of the crude material (water, hexane or dichloromethane), drying under reduced pressure and recrystallized from ethanol (95%) or hot hexane to afford the desired **6b-6e**, **6g-6i**, **6k**. Yields and characterization pattern are described below:

#### *N*-(3,4-dimethoxyphenethyl)-*N*-6-dimethylbenzo[d][1,3]dioxole-5-sulfonamide (6b) LASSBio-959

The title compound was obtained in 96% yield, by alkylation **6a** with methyl iodide, as a beige powder with mp 92–94°C.

^**1**^**H NMR (200 MHz, CDCl**_**3**_**) *δ* (ppm):** 2.41 (s, 3H, Ar- CH_3_), 2.79–2.86 (m, 2H, CH_3_-N, Ar-CH_2_), 3.37 (t, 2H, N-CH_2_, *J* = 8 Hz) 3.85 (s, 3H, O-CH_3_), 3.86 (s, 3H, O-CH_3_), 6.02, (s, 2H, O-CH_2_-O), 6.66–6.79 (m, 4H, H_2_, H_5_, H_6_, H_5’_), 7.36 (s, 1H, H_2’_);

^**13**^**C NMR (50 MHz, CDCl**_**3**_**) *δ* (ppm):** 20.2 (CH_3_-Ar), 34.1 (CH_2_-Ar), 51.2 (N-CH_2_-), 55.7 (O-CH_3_), 55.8 (O-CH_3_), 101.8 (O-CH_2_-O), 110.0 (Ar-C_2’_), 111.3 (Ar-C_5_), 111.9 (Ar-C_2_), 112.0 (Ar-C_5´_), 120.6 (Ar-C_6_), 129.6 (Ar-C_6’_), 130.8 (Ar-C_1_), 133.6 (Ar-C_1’_), 145.5 (Ar-C_3’_), 147.7 (Ar-C_4_), 148.9 (Ar-C_3_), 150.8 (Ar-C_4’_);

**HPLC: λ** 254 nm: 96,6%

#### *N*-(3,4-dimethoxyphenethyl)-*N*-benzyl-6-methylbenzo[d][1,3]dioxole-5-sulfonamide (6c) LASSBio-960

The title compound was obtained in 91% yield, by alkylation **6a** with 1-bromomethylbenzene, as a powder with mp 119–121°C.

**IR (KBr) (cm**^**-1**^**):** 2958, 1452 (ν CH_2_), 2998, 1416 (ν CH_3_), 1257,1040 (ν CO), 1347 (ν C-N);

^**1**^**H NMR (200 MHz, CDCl**_**3**_**) *δ* (ppm):** 2.68 (s, 3H, Ar-CH_3_), 2.68 (t, 2H, CH_2_-CH_2_-Ar, *J* = 8,2 Hz), 3.34 (t, 2H, N-CH_2_-CH_2,_ *J* = 8 Hz), 3.80 (s, 3H, O-CH_3_), 3.84 (s, 3H, O-CH_3_), 4.40 (s, 2H, N-CH_2_-Ar), 6.04 (s, 2H, O-CH_2_-O), 6.47 (d, 1H, H_2_, *J* = 1,8 Hz), 6.57 (dd, 1H, H_6,_ *J* = 1,8 Hz), 6.71 (d, 1H, H_5_, *J* = 3,2 Hz), 6.74 (s, 1H, H_5´,_ *J* = 8 Hz), 7.20–7.34 (m, 5H, Ph), 7.47 (s, 1H, H_2´_);

^**13**^**C NMR (50 MHz, CDCl**_**3**_**) *δ* (ppm):** 20.5 (CH_3_-Ar), 34.1 (-CH_2_-Ar), 48.1 (N-CH_2_-), 51.0 (N-CH_2_-Ar), 55.9 (O-CH_3_), 56.0 (O-CH_3_), 102.0 (O-CH_2_-O), 110.4(Ar-C_2´_), 111.4 (Ar-C_2_), 112.0 (Ar-C_5´_), 120.3 (Ar-C_5´_), 120.7 (Ar-C_6_), 128.0 (Ar-C_2´_), 128.7 (Ar-C_2´´_, Ar-C_6´´_), 130.8 (Ar-C_1_), 131.2 (Ar-C_6´_), 133.7 (Ar-C_1´_), 136.1 (Ar-C_4´´_), 145.8 (Ar-C_3´_), 147.8 (Ar-C_4_), 149.0 (Ar-C_3_), 151.1 (Ar-C_4´_);

**HPLC:** 60/40 acetonitrile/water; **λ** 254nm: 99,3% purity.

#### *N*-(3,4-dimethoxyphenethyl)-*N*-allyl-6-methylbenzo[d][1,3]dioxole-5-sulfonamide (6d) LASSBio-961

The title compound was obtained in 87% yield, by alkylation **6a** with allyl bromide, as a powder with mp 74–76°C. The precipitate was filtered and passed into a flash column for purification (Hexane: EtOAc, 5–10%) followed by recrystallization using hot hexane to obtain **6d**.

**IR (KBr) (cm**^**-1**^**):** 3012 (ν CH), 2917, 1449 (ν CH_2_), 2979, 1417 (ν CH_3_), 1253, 1077 (ν CO);

^**1**^**H NMR (500 MHz, CDCl**_**3**_**) *δ* (ppm):** 2.42 (s, 3H, Ar-CH_3_), 2.76 (t, 2H, -CH_2_-Ar, *J* = 8 Hz), 3.39 (t, 2H, N-CH_2_-CH_2,_ -, *J* = 8 Hz e *J* = 7,5 Hz), 3.82–3.85 (m, 8H, N-CH_2_-CH = CH_2,_ 2O-CH_3_), 5.20–5.24 (m, 2H, CH-CH_2_), 5.67–5.70 (m, 1H, -CH = CH_2_), 6.02 (s, 2H, O-CH_2_-O), 6.60 (s, 1H, H_2_), 6.63 (d, 1H, H_6_, *J* = 8 Hz), 6.67 (s, 1H, H_5´_), 6.74 (d, 1H, H_5,_ *J* = 8 Hz), 7.42 (s, 1H, H_2´_);

^**13**^**C NMR (125 MHz, CDCl**_**3**_**) *δ* (ppm):** 20.3 (Ar-CH_3_), 34.1 (CH_2_-Ar), 47.8 (N-CH_2_-), 49.5 (N-CH = CH_2_), 55.8 (O-CH_3_), 55.9 (O-CH_3_), 102.0 (O-CH_2_-O), 110.3 (Ar-C_2´_), 111.2 (Ar-C_2_), 111.8 (Ar-C_5´_), 112.1 (Ar-C_5_), 119.1 (= CH_2_), 120.6 (Ar-C_6_), 130.6 (Ar-C_6´_), 131.0 (Ar-C_1´_), 133.1 (CH = CH_2_), 145.6 (Ar-C_3´_), 147.6 (Ar-C_4_), 148.9 (Ar-C_3_), 150.9 (Ar-C_4´_);

**HPLC:** 60/40 acetonitrile/water; λ 254nm: 98,6% purity.

#### *N*-(3,4-dimethoxyphenethyl)-*N*-cyclopentyl-6-methylbenzo[d][1,3]dioxole-5-sulfonamide (6e) LASSBio-962

The title compound was obtained in 34% yield, by alkylation **6a** using DMF as a solvent, with cyclopentyl bromide, as a powder (0.04 g).

^**1**^**H NMR (200 MHz, CDCl**_**3**_**) *δ* (ppm):** 1.40–1.89 (m, 8H, cyclopentyl), 2.52 (s, 3H, Ar-CH_3_), 2,77–2,87 (m, 2H, -CH_2_-Ar), 3,29–3,37 (m, 2H, N-CH_2_-CH_2_-), 3.85 (s, 3H, O-CH_3_), 3.87 (s, 3H, O-CH_3_), 3.93–4.02 (m, 1H, N-CH, cyclopentyl), 6.00 (s, 2H, O-CH_2_-O), 6.67–6.81 (m, 4H, H_2,_ H_5,_ H_6,_ H_5´_), 7.46 (s, 1H, H_2´_);

#### *N*-(3,4-dimethoxyphenethyl)-*N*-methylbenzenesulfonamide (6g) LASSBio-982

The title compound was obtained in 89% yield, by alkylation **6f** using acetone as a solvent, with methyl iodide, as a powder with mp 67–68°C.

**IR (KBr) (cm**^**-1**^**):** 3068 (ν CH), 3000 (ν CH_3_), 2938, 1468 (ν CH_2_), 1259,1025 (ν CO);

^**1**^**H NMR (200 MHZ, CDCl**_**3**_**) *δ* (ppm):** 2.75 (s, 3H, N-CH_3_), 2.77–2.85 (m, 2H, CH_2_-CH_2_-Ar), 3.22–3.30 (m, 2H, N-CH_2_-CH_2_), 3.85 (s, 3H, O-CH_3_), 3.86 (s, 3H, O-CH_3_), 6.70–6.82 (m, 3H, H_2_, H_5_, H_6_), 7.49–7.57 (m, 3H, H_3’_, H_5’_, H_4’_), 7.76 (dd, 2H, H_2’_, H_6’_, *J*o = 7 Hz, *J*m = 1,5 Hz).

^**13**^**C NMR (CDCl**_**3**_**) *δ* (ppm):** 34.3 (-CH_2_-Ar), 35.2 (Ar -CH_3_), 51.8 (N-CH_2_-), 56.0 (2C, O-CH_3_), 111.5 (Ar-C_5_), 112.2 (Ar-C_2_), 120.8 (Ar-C_6_), 127.3 (Ar-C_2´_, Ar-C_6´_), 129.1 (Ar-C_3´_, Ar-C_5´_), 130.9 (Ar-C_1_), 132.5 (Ar-C_4’_), 138.0 (Ar-C_1´_), 147.8 (Ar-C_4_), 149.1 (Ar-C_3_).

**HPLC:** 60/40 acetonitrile/water; **λ** 254nm: 96,8% purity.

#### *N*-(3,4-dimethoxyphenethyl)-*N*-allylbenzenesulfonamide (6i) LASSBio-983

The title compound was obtained in 92% yield, by alkylation of **6f** with allyl bromide, as a powder with mp 64–65°C.

**IR (KBr) cm**^**-1**^: 3067 (ν CH), 2940, 1448 (ν CH_2_), 2964, 1420 (ν CH_3_), 1262, 1030 (ν CO);

^**1**^**H NMR (500 MHz, CDCl**_**3**_**) *δ* (ppm):** 2.72 (t, 2H, -CH_2_-Ar, *J* = 8 Hz), 3.28 (t, 2H, N-CH_2_-CH_2,_ -, *J* = 8 Hz), 3.71–3.78 (m, 8H, N-CH_2_-CH = CH_2,_ 2O-CH_3_), 5.05–5.13 (m, 2H, CH-CH_2_), 5.44–5.64 (m, 1H, -CH = CH_2_), 6.59–6.61 (m, 2H, H_2_ e H_6_), 6.69 (d, 1H, H_5_, *J* = 8 Hz), 7.38–7.53 (m, 3H, H_3´,_ H_4´,_ H_5´_), 7.71 (d, 2H, H_2´,_ H_6´,_ *J* = 8 Hz);

^**13**^**C NMR (125 MHz, CDCl**_**3**_**) *δ* (ppm):** 35.1 (CH_2_-Ar), 49.0 (N-CH_2_-), 51.1 (N-CH_2_-CH), 56.0 (2C, O-CH_3_), 111.5 (Ar-C_2_), 112.3 (Ar-C_5_), 119.1 (= CH_2_), 120.9 (Ar-C_6_), 127.2 (Ar-C_3´_, C_5´_), 129.2 (Ar-C_2´,_ C_6´_), 131.5 (Ar-C_4´_), 133.2 (-CH = CH_2_), 140.3 (Ar-C_1´_), 147.9 (Ar-C_4_), 149.1 (Ar-C_3_);

**HPLC:** 60/40 acetonitrile/water; λ 254nm: 97,7% purity.

#### *N*-(3,4-dimethoxyphenethyl)-*N*-benzylbenzenesulfonamide (6h) LASSBio-984

The title compound was obtained in 81% yield, by *N*-alkylation of **6f** with 1-bromomethylbenzene, as a yellow powder with mp 82–84°C.

^**1**^**H NMR (200 MHz, CDCl**_**3**_**) *δ* (ppm):** 2.51 (t, 2H, CH_2_-CH_2_-Ar, *J* = 8 Hz), 3.22 (t, 2H, N-CH_2_-CH_2,_ *J* = 8 Hz), 3.70 (s, 3H, O- CH_3_), 3.75 (s, 3H, O- CH_3_), 4.60 (s, 2H, N-CH_2_-Ph), 6.36 (s, 1H, H_2_), 6.40 (d, 1H, H_6,_ *J* = 8 Hz), 6.63 (d, 1H, H_5,_ *J* = 8 Hz), 7.15–7.24 (m, 5H, Ph), 7.40–7.55 (m, 3H, H_3´,_ H_4´,_ H_5´_), 7.76 (d, 2H, H_2´,_ H_6´,_ *J* = 6 Hz);

^**13**^**C NMR (50 MHz, CDCl**_**3**_**) *δ* (ppm):** 35.1 (CH_2_-Ar), 49.7 (N-CH_2_-), 52.4 (N-CH_2_-Ar), 55.9 (O- CH_3_), 56.0 (O-CH_3_), 111.4 (Ar-C_2_), 112.0 (Ar-C_5_), 120.7 (Ar-C_6_), 127.2 (Ar-C_2´_, Ar-C_6´_), 128.0–128.7 (6C, Ph), 129.8 (Ar-C_3´_, Ar-C_5´_), 131.1 (Ar-C_4´_), 132.6 (Ar-C_1_), 140.2 (Ar-C_1´_), 147.8 (Ar-C_4_), 149.0 (Ar-C_3_);

**HPLC:** 60/40 acetonitrile/water; **λ** 254nm: 99,2% purity.

#### *N*-(3,4-dimethoxyphenethyl)-*N*-methylmethanesulfonamide (6k) LASSBio-1271

The title compound was obtained in 89% yield, by *N*-alkylation **6j** with methyl iodide, as a powder with mp 48–49°C.

**IR (KBr) cm**^**-1**^: 2935, 2849 (C-H), 1468, 1373 (CH_3_), 1325, 1146 (S = O), 1267, 1024 (C-O);

^**1**^**H NMR (200 MHz, CDCl**_**3**_**) *δ* (ppm):** 2.68 (s, 3H, N-CH_3_), 2.75–2.88 (m, 5H,—CH_2_-Ar, S-CH_3_), 3.38 (t, 2H, NH-CH_2_-, *J* = 8 Hz), 3.85 (s, 3H, O-CH_3_), 3.87 (s, 3H, O-CH_3_), 6.75–6.83 (m, 3H, H_2,_ H_5,_ H_6_);

^**13**^**C NMR (125 MHz, CDCl**_**3**_**) *δ* (ppm):** 34.6 (-CH_2_-Ar), 34.9 (N-CH_3_), 36.1 (S-CH_3_), 51.8 (N-CH_2_-), 56.0 (2C, O-CH_3_), 111.4 (Ar-C_2_), 112.0 (Ar-C_5_), 120.8 (Ar-C_6_), 130.8 (Ar-C_1_), 147.9 (Ar-C_4_), 149.1 (Ar-C_3_);

**HPLC:** 60/40 acetonitrile/water; λ 254 nm: 97,2% purity

### Pharmacological Evaluation

#### PDE4 activity evaluation in vitro

PDE4A, 4B, 4C and 4D activities were measured by employing an IMAP TR-FRET protocol (kit from Molecular Devices, Sunnyvale, CA, USA). The enzymatic reactions were carried at room temperature in a 96-well black plate by co-incubating 25 μL of 200 nM FAM-cAMP (R7513), 5 μL of putative inhibitory compounds and 20 μL of the PDE4 isoform dissolved in assay buffer (R7364) for 1 h. All enzymes were obtained from human recombinant sources (MDS PHARMA), whereas the other reagents were purchased from Molecular Devices. Fluorescence polarization intensity was measured at 485 nm excitation and 520 nm emission using a microplate reader, SpectraMax M5 (Molecular Devices, Sunnyvale, CA, USA). PDE4 inhibitors were dissolved in dimethyl sulfoxide (DMSO) at a final concentration of 0.1%. At this condition, the vehicle had no significant effect on PDE4 activity. The concentration of drugs that produced 50% inhibition of substrate hydrolysis (IC_**50**_) was calculated by nonlinear regression analysis from concentration response curves.

#### Animals

Male A/J mice (18–20 g) and male guinea pigs (300–400 g) were obtained from the Oswaldo Cruz Foundation breeding facility and kept in the animal-housing facility of the Laboratory of Inflammation at a controlled room temperature (22°C– 25°C) and with 12 h light/dark cycle. All the procedures involving care and use of laboratory animals in this study were examined and approved by the Animal Ethics Committee of the Oswaldo Cruz Foundation (CEUA—FIOCRUZ—licence 0213–04).

#### Tracheal smooth muscle contraction in vitro

Tracheas from guinea pigs were obtained and prepared as described previously [37]. An initial tension of 1 g was applied to the tracheas for 60 min to obtain a constant resting tension. To confirm the viability of the preparation, the response to carbachol (2.5 μM) was recorded. After washout of carbachol and re-establishement of the baseline resting tension, tissues were preincubated with LASSBio-448 (100 μM) or vehicle (DMSO 0.1%) 15 min before re-exposed to cumulative addition of carbachol (10^−8^–10^−4^ M). The tracheal rings were pretreated 10 min before application of LASSBio-448 with 100 μM SQ22536 (adenylyl cyclase inhibitor). Stimulation-induced isometric contractile responses were measured with a force-displacement transducer (Ugo Basile, Comerio, Italy) and the readout used to assess contractility was obtained by isolated organ data acquisition software (Proto 5; Letica Scientific Instruments, Barcelona, Spain). Contractile responses were expressed as a percentage of the maximal contraction induced by 2.5 μM carbachol.

#### Measurement of cAMP intracellular levels in airway smooth muscle cells

Intracellular cAMP concentrations were assayed in primary cultured guinea-pig tracheal smooth muscle cells as reported [37–38]. Briefly, smooth muscle cells obtained from guinea pig tracheas were cultured in DMEM containing 10% fetal bovine serum, 100 units/ml of penicillin, 100 mg/ml of streptomycin and 2 mM of glutamine for 3 to 7 days. After the third cell splitting, 10^6^ cells/well were grown in 24-well plates. At confluence, smooth muscle cells were washed with PBS and incubated with 100 μM LASSBio-448 or forskolin (adenylyl cyclase activator), in the presence or absence of 100 μM SQ22536 (adenylyl cyclase inhibitor) for 30 min. Lysed cells were collected and the intracellular cAMP was evaluated by means of radioimmunoassay (TRK 432–Cyclic AMP^[3H]^ Biotrak assay system—Amersham Pharmacia Biotech, Buckinghamshire, England) following manufacturer’s guidelines.

#### Sensitisation, allergen challenge and treatment protocol

Sensitization was performed by means of a subcutaneous injection of 50 μg ovalbumin (OVA) (grade V; Sigma-Aldrich, St. Louis, MO) adsorbed to 5 mg of Al(OH)_3_ in 0.2 mL of sterile 0.9% NaCl (saline) at days 0 and 14. Intranasal OVA provocations (25 μg /25 μL saline) were performed at 19 and 20 days post-sensitization under isofluorane volatile anaesthesia (Cristalia, São Paulo, Brazil). Alternatively, sensitization as described above were done at days 0 and 7, while the OVA provocations were carried out at days 14, 21, 28 and 35 [39]. Negative control groups were represented by sensitized mice in which allergen was replaced by saline as a challenge. Treatments were done orally 1 h before provocations. Test compounds were dissolved in saline containing 0.2% Tween 80. All solutions were freshly prepared immediately before use. Analyses were performed 24 h after the last provocation.

#### LPS-induced inflammation

A/J mice were anesthetized with isoflurane aerosol and then challenged with LPS (25μg/25 μL) or phosphate buffered solution (PBS) by intranasal instillation as reported [37]. The analyses were performed 24 h after stimulation. Treatment with LASSBio-448 (2.5–10 mg/kg/mice) or cilomilast (1 mg/kg) was performed orally, 1 h before LPS exposure. LASSBio-448 was dissolved in 0.2% Tween 80, while cilomilast was dissolved in 1 M NaOH and further neutralized with 1 N HCl, before adjusting the final volume with 0.9% NaCl solution (saline). The substances were dissolved immediately before use.

#### Airway hyper-reactivity using non-invasive barometric plethysmography

Using barometric whole body plethysmography (Buxco Research System, Wilmington, NC) as described [38], we measured the enhanced pause responses (Penh) in conscious, spontaneously breathing mice following appropriate provocations. Aerosolized phosphate-buffered saline (PBS) and increasing methacholine concentrations (3, 6, 12 mg/ml) were nebulized through an inlet of the individual chambers for 2.5 min, and Penh readings were recorded for 5 min following each nebulization. Penh averages were obtained at 24 h after the last OVA provocation.

#### Airway hyper-reactivity using invasive barometric plethysmography

Airflow and transpulmonary pressure were recorded with a Buxco pulmonary mechanics processing system (Buxco Electronics, Sharon, CT), which calculated resistance (cmH_2_O.s/ml) and dynamic lung compliance (mL/cmH_2_O) in each breath cycle. Elastance was calculated as the inverse of compliance values [39, 40]. Mice were anesthetized with nembutal (60 mg/kg), and the neuromuscular activity was blocked with bromide pancuronium (1 mg/kg). Animals were allowed to stabilize for 5 min and increasing concentrations of methacholine (3–27 mg/mL) were aerosolized for 5 min each. Baseline pulmonary parameters were assessed with aerosolized phosphate buffer solution (PBS).

#### Eosinophil peroxidase activity (EPO) in lung tissue

The EPO activity present in lung tissue was determined with a colorimetric assay as described by Strath et al. (1985) [41], with minor modifications. Briefly, the lungs were cannulated and perfused with saline/EDTA (20 mM). Then, lung samples were homogenized in buffer solution containing 0.5% HBSS (Hank´s balanced solution, pH 7.4; Sigma Chemical Co.) using a tissue homogenizer (T25 ultra-Tirrax). Samples were centrifuged at 1300 x*g* for 10 min at 4°C. Red blood cells were removed by hypotonic and hypertonic lysis. The suspended cells were then centrifuged again at 1300 x*g* for 10 min at 4°C. The pellet was then suspended in a solution of HBSS containing 0.5% of HTAB (hexadecyltrimethylamonium bromide; Sigma Chemical Co.) (pH 7.4). This suspension was subjected to heat shock for lysing the cells in three cycles freeze/thawed. At the end, the supernatants were collected, centrifuged at 12000 rpm for 15 min at 4°C and assayed spectrophotometrically for EPO determination. Fifty μl of the samples were placed, and then 50 μl of the substrate (1.5 mM o-phenylenediamine and 6.6 mM hydrogen peroxide in 0.05 mM Tris-HCl buffer, pH 8.0) was added and, after a 30 min incubation at room temperature, 50 μl of H_2_SO_4_ (4M) was added to stop the reaction. The optical density reading was performed on the SpectraMax M5 spectrophotometer (Molecular Devices) at a wavelength of 492 nm.

#### Myeloperoxidase (MPO) activity assay

The MPO activity present in the lung tissue was colorimetrically determined as described by Hirano (1996) [42], with minor changes. Briefly, after lung perfusion with 20 mM EDTA in 0.9% saline, lung samples were homogenized in Hank´s balanced solution (HBSS) at pH 7.4. The homogenates were centrifuged for 10 min at 1300 x*g* and 4°C. Red blood cells were removed by hypotonic and hypertonic lysis and suspended cells were re-centrifuged as described above. The pellet was then suspended in HBSS at pH 7.4 containing 0.5% hexadecyltrimethyl ammonium bromide (HTAB). The homogenates were centrifuged for 30 min at 12500 x*g* and 4°C. The supernatants were then collected and incubated with 0.167 mg/mL of ortho-dianisidine dihydrochloride for 15 min at 37°C, after which 0.0006% H_**2**_O_**2**_ in 50 mM phosphate buffer at pH 6 was added. The reaction was stopped 10 min later by adding 50 μL of 1% sodium azide. The measurement was done at 460 nm using the SpectraMax M5 microplate reader (Molecular Devices).

#### Brochoalveolar lavage (BAL)

Twenty-four hours after the last challenge the animals were killed by an over dose of thiopental (200 mg/kg, i.p.) and bronchoalveolar lavage (BAL) was performed three times by intratracheal instillation of 500 μL of PBS and EDTA (10 mM) through a trachea cannula and gently aspirating the fluid. Samples were centrifuged at 300 x*g* for 10 min, and cell pellets were suspended in 0.25 mL PBS. Total leukocyte numbers were measured in Neubauer chambers using light microcopy after diluting the samples in Türk solution (2% acetic acid). Differential cell counts were performed with cytospin smears using the May-Grünwald Giemsa method. At least 100 cells were counted per slide under light microscopy, and they were differentiated according to standard morphological criteria. The percentages of differential cells were multiplied by total cells number to obtain the number of different cells populations [38–40].

#### Cytokines quantification

Right lungs tissue samples from saline- and OVA-exposed mice were were homogenized in PBS containing 0.05% Triton X-100 and a protease inhibitor cocktail (Complete®, Hoffmann-La Roche Ltd, Basel, Switzerland). After centrifugation at 13000x*g* for 10 min and 4°C, the levels of IL-13, IL-5, IL-4 and eotaxin-2 were quantified in the supernatant, using commercially available ELISA kits (DuoSet; R&D Systems, Minneapolis, MN, USA). The results were expressed as picograms of cytokine per right lung.

#### Duration of anesthesia

This assessment was carried out as previously reported [43–44]. Mice of strain A/J were treated orally with rolipram, cilomilast, LASSBio-448 or vehicle and then anesthetized with a combination of xylazine (10 mg/kg) and ketamine (70 mg/kg), administered as a single hindlimb intramuscular injection 30 min post-treatment. The animals were placed in dorsal position and their spontaneous return to the prone position was used as an endpoint to determine anesthesia duration. Both rolipram and LASSBio-448 were freshly dissolved in Tween 80 0.3% immediately before use.

### Molecular Docking

Docking studies with GOLD software version 5.2 [32], using goldscore scoring function were carried out to get insights on the detailed interactions of LASSBio-448 with PDE4 isoforms A, B, C and D. Marvin version 6.2.1, 2014, ChemAxon, was used for drawing, displaying and characterizing the ligand’s chemical structures (http://www.chemaxon.com).

The crystal structures of the catalytic domains of human PDE4 isoforms were retrieved from the protein Data Bank-PDB, with the ID Codes 3I8V, 4KP6 and 1Y2K (PDEs A, B and D, respectively) [45–49].

Since the available crystal structure of unliganded PDE4C2 isoform has a considerable degree of disorder in the region where the conserved Glutamine is located (PDB code 2QYM) [45], we have built a homology model using the FASTA sequence of PDE4C and the structure of PDE4D (best template found) at a high resolution (ID Code 3G4I), chain A [49] in the automated mode in Swiss-Model [50–51].

The set of amino acid residues selected as the binding site to perform docking studies was determined by a distance of 10 Å from the conserved NE2 of Gln369 (PDE4D numbering). All catalytic water molecules have been kept during docking procedures, since it has been shown in the literature that they are important in the binding process [48–49]. Metal ions were included as part of the active site as well. At last, ligand-protein interactions’ analysis have been performed with Hermes, available as part of GOLD docking suite and Pymol v. 0.99, the latter of which has also been used to build molecular surfaces.

### Statistical analysis

All data are presented as means ± standard error of the mean (SEM) and statistical analysis involving two groups was done, with Student´s t test, whereas ANOVA followed by the Newman-Keuls-Student´s t test were used to compare more than 2 groups. *P* values of 0.05 or less (two-tail test) were considered significant.

## Results and Discussion

### Chemistry

**Fig 3:**
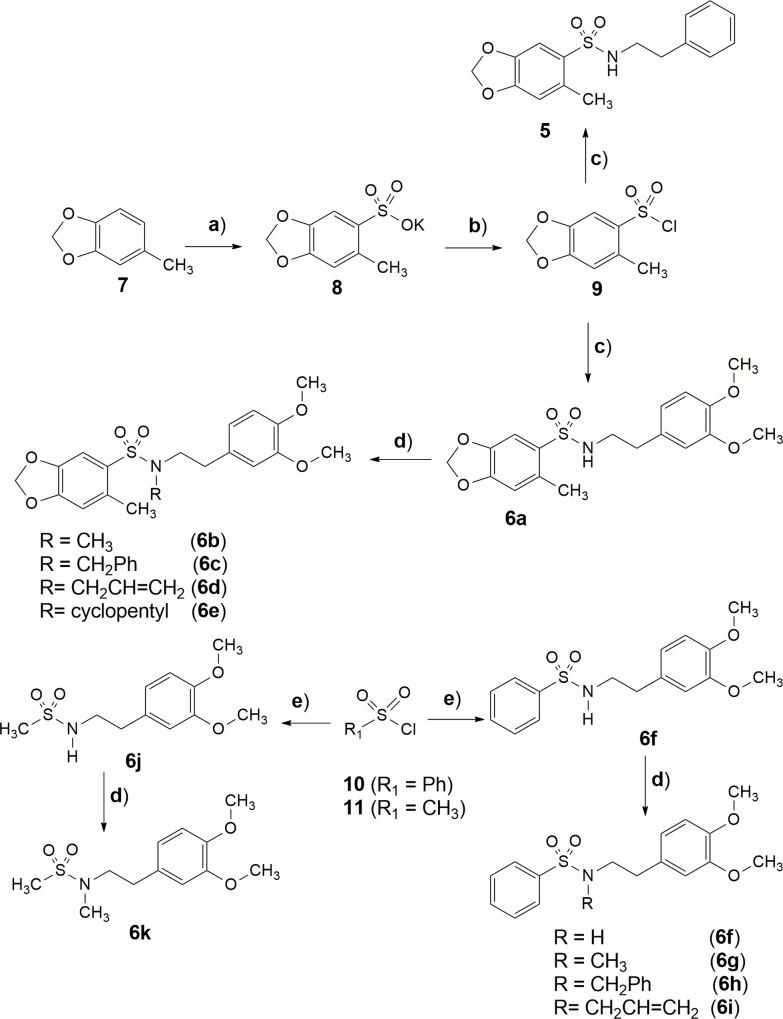
Synthesis sulfonamide derivatives. Reagents and conditions: a) 1) H_2_SO_4_ /Ac_2_O /AcOEt, 0°C, 2 h; 2) AcOK / EtOH, 25°C, 30 min, 93%. b) SOCl_2_, DMF, 75°C, 4 h, 92%. c) CH_2_Cl_2_, Et_3_N, 2-(3,4-dimethoxyphenyl)ethanamine (**6a**) or 2-phenylethanamine (5), 25°C, 2–2.5 h, 70%-81% respectively. d) K_2_CO_3_, acetone, RX (X = I, Br), (**6b-6e, 6g-6i** and **6k**), 40°C, 1.5 h, 34–96%. e) CH_2_Cl_2_, Et_3_N, 2-(3,4-dimethoxyphenyl)ethanamine (**6f, 6j**) 25°C, 2–2.5 h, 53%-83% respectively.

The synthesis of the target sulfonamides **5** and **6a** was accomplished in a multistep linear synthesis. The process starting from a regiosselective sulfonation of 5-methylbenzo [*d*][1,3] dioxole (**7**), followed by chlorination of the corresponding salt (**8**) under the conditions depicted in Fig 3. The key intermediate **9** was subjected to condensation reactions with 2-phenylethylamine and 3,4-dimethoxyphenethylamine to obtain **5** and **6a**, respectively. Compounds **6f** and **6j** were synthetized in high yields by condensation between the commercial chlorosulfonic derivatives (**10** and **11**) and the 3,4-dimethoxyphenethylamine, in the presence of triethylamine as base and dichloromethane as solvent (Fig 3). The homologous series (**6b-i** and **6k**) were obtained by *N*-alkylation of compounds **6a**, **6f** and **6j**, using the methodology previously described by Barco and coworkers [12]. All compounds were characterized by NMR ^1^H, NMR ^13^C, infrared and mass spectrometry. The relative purity was determined by HPLC analysis. The comparative physico-chemistry properties of rolipram, prototype **4** and its analogues **5** and **6a-k** were calculated *in silico* using the Program ACD/Percepta 14.0. As demonstrated in Table 1 no violations to Lipinski’rule of five were observed, anticipating a probable orally administered drug profile [13].

**Table 1:** Physico-chemistry properties of rolipram (1), prototype 4 and its sulfonamide analogues 5 and 6a-k, calculated using the Program ACD/Percepta 14.0.

Compounds	MW	cLogP	HDB	HAB	TPSA	LogD_7.4_	pKa	Solubility (mg/ml)
**1** (Rolipram)	275.34	1.87	1	4	47.56	1.8	15.7	0.35
**4**	319.38	2.88	0	5	64.22	2.9	N/A	0.01
**5** (LASSBio-964)	319.38	2.87	1	5	73.01	2.9	11.8	0.009
**6a** (LASSBio-448)	379.43	2.49	1	7	91.47	2.5	11.4	0.02
**6b** (LASSBio-959)	393.46	2.73	0	7	82.68	2.7	N/A	0.008
**6c** (LASSBio-960)	469.55	3.58	0	7	82.68	3.6	N/A	0.0003
**6d** (LASSBio-961)	419.49	3.02	0	4	82.68	3.0	N/A	0.01
**6e** (LASSBio-962)	447.55	3.48	0	7	82.68	3.5	N/A	0.003
**6f** (LASSBio-965)	469.55	3.58	0	7	73.01	2.7	11.1	0.0003
**6g** (LASSBio-982)	355.42	3.23	0	5	64.22	3.2	N/A	0.03
**6h** (LASSBio-984)	411.51	4.68	0	5	64.22	4.7	N/A	0.0004
**6i** (LASSBio-983)	361.46	3.15	0	5	64.22	3.2	N/A	0.02
**6j** (LASSBio-985)	259.32	1.24	1	5	73.01	1.2	11.0	1.49
**6k** (LASSBio-1271)	273.35	1.53	0	5	64.22	1.5	N/A	1.37

N/A = Not Applied.

### Biology

#### In vitro studies

The inhibitory activities of the new sulfonamide derivatives were evaluated for their selectivity against the four isoforms of human PDE4, i.e. PDE4A, PDE4B, PDE4C, and PDE4D, using an IMAP-FP protocol. Compounds (**5** and **6a-k**) were tested at a screening concentration of 1 μM using rolipram as standard. Those inhibiting 30% or more were selected for further potency investigation. As indicated in Table 1, rolipram showed inhibitory activity against all the four PDE4 isoenzymes, while sulfonamide **6a** was able to inhibit PDE4A, PDE4B and PDE4C activities. Compounds **5**, **6b**, **6d**, **6g**, **6i** and **6j** showed only a marginal activity against PDE4D (Table 2).

**Table 2:** PDE4 Inhibition of rolipram and sulfonamides 5 and 6a-k.

Compounds	PDE4A1A	PDE4B1	PDE4C	PDE4D3
**1** (Rolipram)	71.0 ± 0.4	60.0 ± 1.2	67.0 ± 1.3	50.0 ± 0
**5** (LASSBio-964)	Inactive	Inactive	3.9 ± 2.9	33.5 ± 4.5
**6a** (LASSBio-448)	36.5 ± 0.7	31.0 ± 1.1	33.7 ± 0.4	26.5 ± 0.7
**6b** (LASSBio-959)	Inactive	Inactive	5.6 ± 1.0	42.4 ± 3.5
**6c** (LASSBio-960)	Inactive	Inactive	2.0 ± 0.8	21.6 ± 4.5
**6d** (LASSBio-961)	Inactive	Inactive	6.3 ± 4.1	40.4 ± 4.5
**6e** (LASSBio-962)	Inactive	Inactive	7.6 ± 1.1	23.6 ± 2.2
**6f** (LASSBio-965)	Inactive	Inactive	9.7 ± 3.3	19.3 ± 4.0
**6g** (LASSBio-982)	Inactive	Inactive	4.1 ± 0	37.4 ± 3.9
**6h** (LASSBio-984)	Inactive	5.0 ± 0.6	9.8 ± 1.7	14.3 ± 5.4
**6i** (LASSBio-983)	Inactive	Inactive	9.7 ± 0.8	67.6 ± 2.6
**6j** (LASSBio-985)	Inactive	12.6 ± 2.1	16.3 ± 1.0	62.9 ± 3.8
**6k** (LASSBio-1271)	Inactive	Inactive	Inactive	Inactive

All compounds were used at the concentration of 1 μM. Percentual values of inhibition of PDE4 isoenzymes were represented as mean ± SEM from 3 distinct experiments. Values below 2% of inhibition were indicated as inactive.

PDE4A, B and D are the isoenzymes expressed in human leukocytes and they have a central role in inflammatory diseases [14, 15]. Recent review shows that PDE4C is not normally found in inflammatory cells and it is not related with inflammatory response [14, 15]. Regarding the most important side effect of PDE4 inhibitors, Robichaud and coworkers [16] have demonstrated that emesis is produced as a result of PDE4D inhibitory activity. This isoenzyme is one of the four PDE4 genes products present in the brainstem. Studies carried out with PDE4D-knockout mice confirmed that emesis is strongly linked to the PDE4D inhibition [17]. Therefore, the analysis of the results showed in Table 2 allowed the selection of sulfonamide **6a** (LASSBio-448) to further investigation. The comparative potency of rolipram and LASSBio-448 (**6a**) against PDE4A, B, C and D was established. As demonstrated in Table 3, sulfonamide **6a** (LASSBio-448) was able to inhibit recombinant PDE4A, PDE4B, PDE4C and PDE4D with IC_50_ values of 0.7 μM, 1.4 μM; 1.1 μM and 4.7 μM, respectively. While, rolipram inhibited PDE4A, PÞ4B, PDE4C and PDE4D with IC_50_ values of 0.3 μM, 0.9 μM; 0.9 μM and 0.6 μM, respectively. The comparison between the selective inhibitory index (SI) against PDE4D/PDE4A or PDE4D/PDE4B for **6a** and rolipram revealed a more favorable profile for sulfonamide **6a** (LASSBio-448). This compound showed a SI of 6.7 (PDE4D/PDE4A) and 3.3 (PDE4D/PDE4B), while rolipram had a SI of 2.0 (PDE4D/PDE4A) and 0.7 (PDE4D/PDE4B), suggesting that LASSBio-448 (**6a**) could be less pro-emetic than the standard rolipram.

**Table 3:** PDE4 recombinant isoform inhibition (IC_50_, μM) for sulfonamide 6a (LASSBio-448) and rolipram.

Recombinant enzyme	6a (LASSBio-448) IC_50_^a^ (μM)	Rolipram IC_50_^a^ (μM)
PDE4A	0.7 ± 0.07	0.3 ± 0.03
PDE4B	1.4 ± 0.09	0.9 ± 0.04
PDE4C	1.1 ± 0.13	0.9 ± 0.02
PDE4D	4.7 ± 0.09	0.6 ± 0.10

^a^The IC_50_ was calculated by nonlinear regression and represents the mean ± SEM value of three measurements.

The selectivity profile of compound **6a** (LASSBio-448) toward the human PDE1, PDE2, PDE5-PDE8, PDE10 and PDE11 was evaluated by CEREP. At concentration of 10 μM, this compound was unable to inhibit PDE1B, PDE2A and, PDEs5-11 (data not shown). At the same concentration (10 μM) LASSBio-448 also failed to inhibit PDE3 activity, under conditions where milrinone was active (IC_50_ = 3.2 μM, n = 4).

It is well established that increased intracellular levels of cAMP mediates smooth muscle relaxation [6]. We noted that **6a** (LASSBio-448), at 100 μM, inhibited tracheal contraction induced by cumulative addition of increasing concentrations of carbachol (S1A Fig), and up-regulated cAMP intracellular levels in cultured tracheal smooth muscle cells, reaching levels comparable to that elicited by the adenylyl cyclase activator forskolin (S1B Fig). Moreover, pre-treatment with 100 μM of SQ22,536, a standard adenylyl cyclase inhibitor, clearly abrogated the cAMP elevating effect (S1B Fig), as well as the relaxant effect, caused by LASSBio-448 (S1A Fig) in our conditions. Altogether, these in vitro findings provide evidence that **6a** (LASSBio-448) has bronchodilator and protective effect in addition to the anti-PDE4 effect.

Further, the comparative in silico ADME profile of LASSBio-448 (**6a**) and rolipram was established as depicted in Table 4. Both compounds were predicted to be highly permeable (Caco-2), highly absorbed (HIA) and foreseen with great oral bioavailability (F%). The in silico prediction of their ADME profile also anticipate their ability to penetrate in central nervous system (CNS score), and a moderate and strong plasma protein binding (PPB) profile for rolipram and LASSBio-448, respectively, resulting in differences in their calculated volume of distribution (Vd). The metabolic stability of both compounds was predicted as undefined (data not shown).

**Table 4:** Comparative ADME properties of rolipram (1) and LASSBio-448 predicted *in silico* using the Program ACD/Percepta 14.0.

Compounds	Caco-2	HIA (%)	F% (oral)	Vd	PPB(%)	CNS
**1** (Rolipram)	*P*_*e*_ = 180 x10^-6^ cm/s	100	99%	1.4 L/Kg	63	-2.06
**6a** (LASSBio-448)	*P*_*e*_ = 211x10^-6^ cm/s	100	99%	1.8 L/Kg	87	-2.54

#### In vivo studies

**Fig 4:**
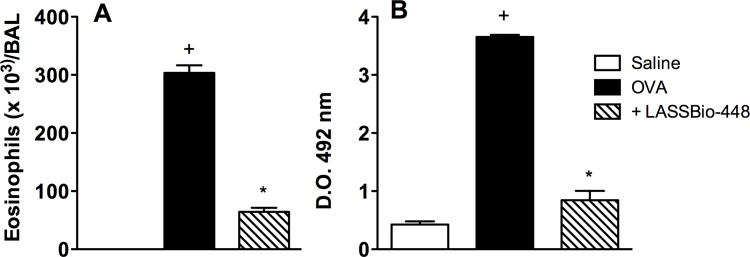
Effect of LASSBio-448 on ovalbumin (OVA)-induced infiltration of eosinophils in the BAL fluid (A) and lung tissue (B) from A/J mice. Animals were sensitized on days 0 and 7 and then challenged with OVA (25 μg/mouse) or saline on days 19 and 20. Treatment with LASSBio-448 (100 mg/Kg, oral), was given 1 h before each OVA challenge, and analyses were performed 24 h after the last stimulation. Values represent mean ± SEM from at least 3 animals. +*P*<0.05 as compared to saline-challenged group; *P<0.05 as compared to OVA-challenged group.

In asthmatics, airway hyper-reactivity and other pathological features of the disease are suggested to be associated in a causative manner with lung eosinophilic infiltration [18, 19]. Not for nothing, various new anti-asthma therapies based in targeting eosinophils and their products are in development [20]. Using a short-term murine model of asthma, characterized by two allergen provocations at days 19 and 20 post-sensitization [21], we wanted to know, as a first approach, whether **6a** (LASSBio-448) might prophylactically inhibit allergen-induced eosinophilic infiltration. We found that LASSBio-448 (100 mg/Kg), administered orally 1 h before challenge, clearly prevented the up-regulation of eosinophil levels in bronchoalveolar lavage fluid, as shown by eosinophil enumeration (Fig 4A) and lung tissue homogenates, attested by EPO colorimetric assay (Fig 4B), in samples obtained 24 h after allergen provocation. PDE4A, PDE4B and PDE4D isoenzymes are expressed in human eosinophils, being quite plausible that increased PDE4 function might account, at least partially, for the pathogenesis of asthma [22]. In fact, PDE4 inhibitors are very efficacious in inhibiting the activation and release of a range of pro-inflammatory mediators by eosinophils, including cytokines, reactive oxygen species and cationic proteins [23, 24]. Additionally, PDE4 inhibitors have been shown to reduce the influx of eosinophils to the lung of allergen-challenged animals, whilst also reducing the bronchoconstriction and elevated bronchial responsiveness [23, 25, 26]. Therefore, it is not unlikely that the protective effect LASSBio-448 (**6a**) upon allergen-induced eosinophil recruitment is, in some extent, dependent on the blockade of PDE4 isoenzymes.

The biological activity exerted by eosinophils is primarily attributable to the release of their granular content, including EPO, eosinophil cationic protein (ECP) and major basic protein (MBP), as well as pro-inflammatory cytokines and chemokines [20]. In line with this concept, eosinophils and eosinophil-derived products have been found in high amounts in bronchial mucosa, sputum and BAL effluent of asthmatics, and appear to be directly associated with asthma severity and characteristic features including mucus hyper-secretion, extracellular matrix deposition and airway hyper-reactivity [27–30].

**Fig 5:**
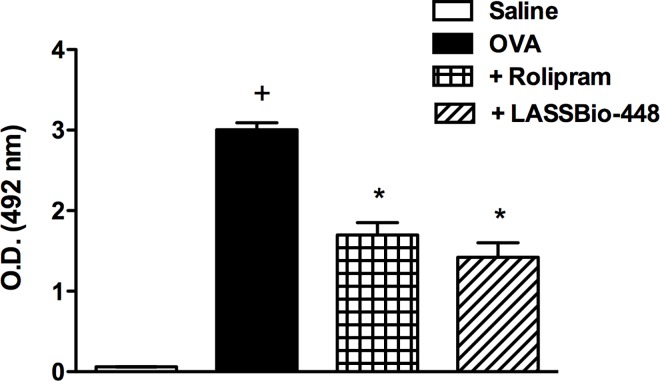
Effect of rolipram and LASSBio-448 on ovalbumin (OVA)-induced infiltration of eosinophils in the lung tissue from A/J mice. Animals were sensitized on days 0 and 7 and then challenged with OVA (25 μg/mouse) or saline on days 14, 21, 28 and 35. Animals were treated with rolipram (10 mg/Kg, oral) or LASSBio-448 (100 mg/Kg, oral) on days 26 and 22, 1 h before OVA challenge, and analyses performed 24 h after the last challenge. Values represent mean ± SEM from at least 7 animals. + P<0.05 as compared to saline-challenged group; *P<0.05 as compared to OVA-challenged group.

We then investigated what would be the impact of the oral treatment with LASSBio-448 (**6a**) on ongoing asthma changes. For this purpose, an alternative murine model of asthma was employed, characterized by 4 weekly ovalbumin provocations done at days 14, 21, 28 and 35 post-sensitization. As illustrated in the S2 Fig, an intense peribronchiolar leukocyte infiltration, accompanied by mucus exacerbation and increased production of extracellular matrix could be detected already after the third allergen provocation, compared with sham-challenged mice. Under this condition, a state of airway hyper-reactivity (AHR) to aerolized methacholine was already noted. Values of Penh response (an indirect measure of lung resistance) increased from 0.61 ± 0.04 to 1.18 ± 0.12 at the concentration of 3 mg/ml, from 1.11 ± 0.11 to 1.63 ± 0.09 at the concentration of 6 mg/ml, and from 1.84 ± 0.1 to 2.75 ± 0.28 at the concentration of 12 mg/ml of methacholine (mean ± SEM) (*p*<0.05, n = 6). Results were interpreted as indicating that, as performed at the third and fourth weeks of allergen challenges, the treatment would encounter an ongoing asthmatic process. Actually, it is in this context that the exposure to either LASSBio-448 (100 mg/Kg, oral) or rolipram (10 mg/kg, oral) has been shown to impair the progress of allergen-induced eosinophil accumulation in the lung tissue, as pointed out by the colorimetric quantification of eosinophil peroxidase (Fig 5).

**Fig 6:**
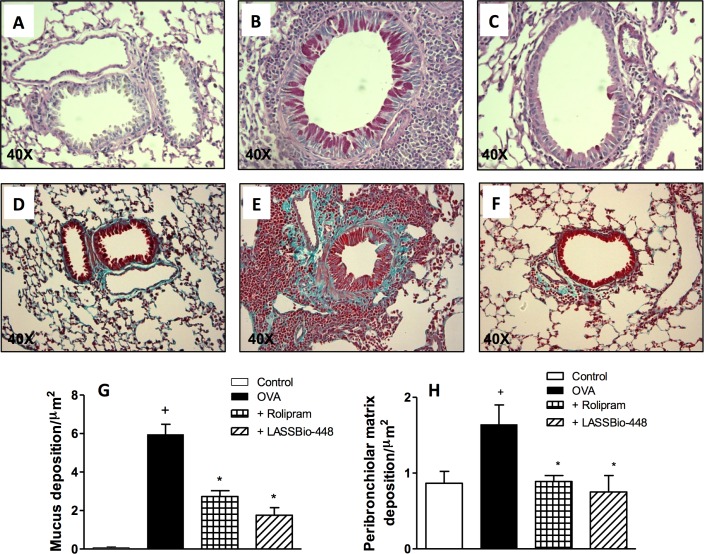
Effect of rolipram and LASSBio-448 on ovalbumin (OVA)-induced mucus production (upper panels) and subepithelial fibrosis (lower panels) in the lungs from A/J mice. Animals were sensitized on days 0 and 7 and then challenged with OVA (25 μg/mouse) or saline on days 14, 21, 28 and 35. Animals were treated with rolipram (10 mg/Kg, oral) or LASSBio-448 (100 mg/Kg, oral) on days 26 and 22, 1 h before OVA challenge, and analyses performed 24 h after the last challenge. The analyses were performed in sensitized mice challenged with saline (A, D), OVA (B, E) and OVA treated with LASSBIo-448 (C, F). Morphometric analyses are seen in (G) mucus production and (H) subepithelial fibrosis. Slides were stained periodic acid-Schiff (upper panels) and Gomori trichrome (lower panels). Values represent mean ± SEM from at least 7 animals. + *P*<0.05 as compared to saline-challenged group; *P<0.05 as compared to OVA-challenged group.

**Fig 7:**
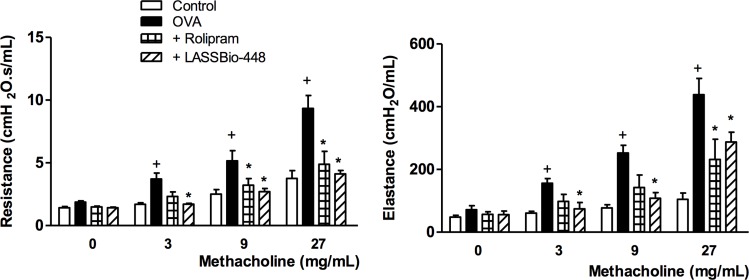
Effect of rolipram and LASSBio-448 on ovalbumin (OVA)-induced changes in lungs from A/J mice. Airway responsiveness was measured by changes in airway resistance (A) and elastance (B) induced by increasing concentrations of methacholine, 24 h after the last ovalbumin or saline challenge. Animals were sensitized on days 0 and 7 and then challenged with OVA (25 μg/mouse) or saline on days 14, 21, 28 and 35. Animals were treated with rolipram (10 mg/Kg, oral) or LASSBio-448 (100 mg/Kg, oral) on days 26 and 22, 1 h before OVA challenge, and analyses performed 24 h after the last challenge. Values represent mean ± SEM from at least 7 animals. + *P*<0.05 as compared to saline-challenged group; *P<0.05 as compared to OVA-challenged group.

Moreover, as revealed by histologic evaluations of lungs from untreated (Fig 6A–6E) and treated mice (Fig 6C and 6F), mucus exacerbation (Fig 6B) and extracellular matrix deposition (Fig 6E) were also sensitive to the interventional treatment with LASSBio-448. Quantitative data on the effects of LASSBio-448 (**6a**) and rolipram are shown in Fig 6H and 6G, concerning mucus and extracellular matrix production, respectively. These treatments were also equally effective in inhibiting airway hyper-reactivity to methacholine, with regard to the functional parameters of airway resistance (Fig 7A) and lung elastance (Fig 7B).

**Fig 8:**
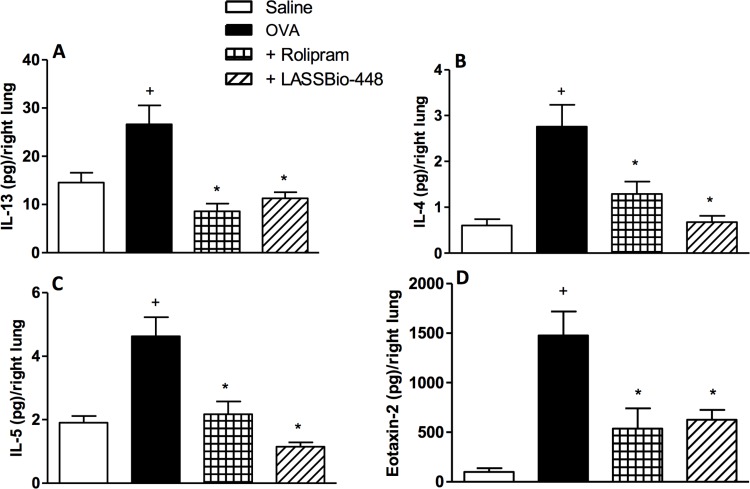
Effect of rolipram and LASSBio-448 on ovalbumin (OVA)-induced cytokine production in the lungs of A/J mice. IL-13 (A), IL-4 (B), IL-5 (C) and eotaxin-2 (D). Animals were sensitized on days 0 and 7 and then challenged with OVA (25 μg/mouse) or saline on days 14, 21, 28 and 35. Animals were treated with rolipram (10 mg/Kg, oral) or LASSBio-448 (100 mg/Kg, oral) on days 26 and 22, 1 h before OVA challenge, and analyses performed 24 h after the last challenge. Values represent mean ± SEM from at least 7 animals. + *P*<0.05 as compared to saline-challenged group; *P<0.05 as compared to OVA-challenged group.

In addition, portions of the lung tissue (right lung), collected at 24 h after the last allergen challenge, were homogenized to evaluate pro-inflammatory cytokine levels. In LASSBio-448 (**6a**) or rolipram-treated mice, compared to vehicle treated ones, a good correlation between the reduction in the levels of pivotal inflammatory mediators, such as IL-4 (Fig 8A), IL-5 (Fig 8B), IL-13 (Fig 8C) and eotaxin-2 (Fig 8D), and down-regulation of crucial asthma features assessed in this model was observed. Taken together, these results suggest that LASSBio-448 (**6a**), similarly to rolipram, not only can prevent the establishment of inflammatory and adverse remodeling changes as applied prophylactically, but also down-regulates these changes during ongoing allergic provocation as administered therapeutically.

**Fig 9:**
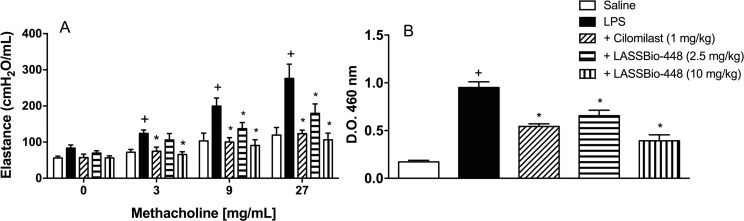
Effect of LASSBio-448 and cilomilast on lung pathological changes caused by LPS in mice. LASSBio-448 (2.5 and 10 mg/kg, oral) or cilomilast (1 mg/kg, oral) were given 1 h before challenge (LPS, 25 μg/25 μL), and analyses on airway hyper-reactivity (A) and neutrophil infiltration, attested by MPO activity of lung tissue samples (B), were carried out 24 h post challenge. Values represent mean ± SEM from at least 7 animals. + *P*<0.05 as compared to vehicle-stimulated group; *P<0.05 as compared to LPS-stimulated mice.

In another setting of experiments, we found that the oral administration of LASSBio-448 (2.5 and 10 mg/kg), 1 h before provocation, dose-dependently prevented LPS-induced airway hyper-reactivity 24 h post-challenge, as attested by quantification of lung elastance values (cmH_2_O/mL) following exposure to aerolized methacholine (3–27 mg/ml) (Fig 9A). This efficacy seems to be accounted for by a marked reduction in the neutrophil infiltration into the lung tissue (Fig 9B). In our conditions, the magnitude of blockade in changes in both functional and inflammatory parameters were similar to that evidenced by cilomilast (1 mg/kg, oral) (Fig 9), suggesting a more comparable effectiveness and potency of LASSBio-448 concerning a reference compound in the current therapy.

**Fig 10:**
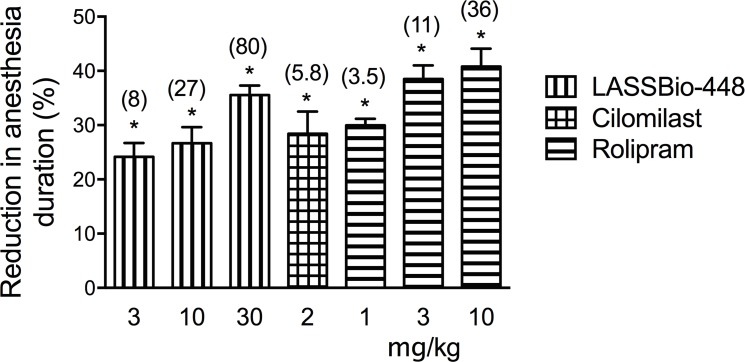
Reduction by LASSBio-448, rolipram or cilomilast in duration of the ketamine/xylazine anesthesia (%). Mice of the strain A/J were injected with ketamine/ xylazine solution and then treated orally with LASSBio-448 (3, 10 and 30 mg/kg), rolipram (1, 3 and 10 mg/kg) or cilomilast (1 mg/kg). Values represent mean ± SEM from at least 7 animals. Figures in brackets shown in the top of each column are correspondent doses expressed in μmol/kg. *P<0.05 as compared to vehicle-treated group.

While exploring the therapeutic window of LASSBio-448 (**6a**), we have done investigations into the emetic potential of this compound in comparison to rolipram and cilomilast. For that purpose, we used a well-established pharmacological approach, in which the emetic response associated with PDE4 inhibitors is indirectly attested by the ability of these agents to shorten the duration of α2-adrenoceptor-mediated anesthesia, a behavioral correlate of emesis in mice, rats and ferrets [16, 17, 31]. In A/J mice, the duration of anesthesia induced by the combined administration of xylazine (10 mg/Kg) and ketamine (70 mg/Kg), as shown by the loss of righting reflex, was 51.7 ± 1.8 min (mean ± SEM; n = 7). Our findings confirmed the anesthesia reversing property of rolipram [31], since as administered orally at 3.5, 11 and 36 μmol/Kg, it yielded sleep time reductions of 30%, 38.5% and 40.8% respectively (10). LASSBio-448 also reduced the duration of anesthesia in 24.1%, 26.6% and 35.5%, following oral doses of 11, 27 and 80 μmol/Kg respectively, suggesting a slight but significant lower pro-emetic activity of the sulfonamide compound (Fig 10). Furthermore, a 28.4% (n = 5) (P<0.01) reduction in the sleep time, induced by xylasine/ketamine anesthesia, was obtained after treatment with cilomilast (5.8 μmol/kg, oral), which is about the one fifth of the equieffective dose of LASSBio-448. One potential explanation for this difference concerning rolipram and cilomilast is probably related to the lower inhibitory effect of LASSBio-448 upon PDE4D as pointed out in our *in vitro* experiments.

### Molecular Docking

In order to establish the putative binding-model of sulfonamide **6a** (LASSBio-448) in the active site of human PDE4 isoenzymes, docking studies were performed using GOLD software version 5.2 (License: G/414/2006) [32]. PDE4D numbering has been used in order to facilitate visual analysis of the docking poses.

**Fig 11:**
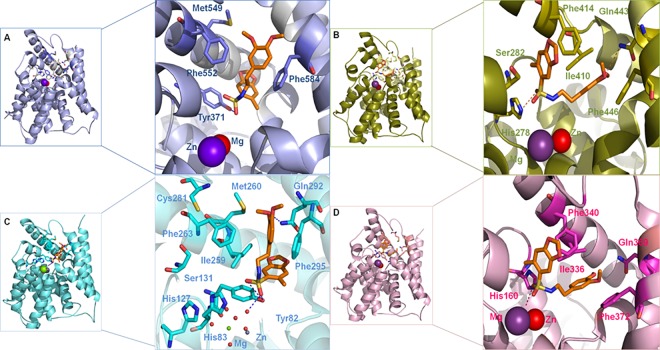
Top poses of LASSBio-448 (orange carbon atoms) with PDE4A (A), PDE4B (B), PDE4C (C) and PDE4D (D) obtained with GOLD 5.2 software. Hydrogen atoms have been omitted for clarity. Hydrogen bonds are in dashed lines. PDE4D numbering has been used.

Docking analysis of the putative interactions of LASSBio-448 (**6a**) with PDE4A-D showed similar poses in the active site of PDE4A and PDE4C, but different orientations in PDE4B and D (Fig 11). However, a common feature seen in the four top poses of LASSBio-448 (**6a**) with the studied isoforms is a π-stacking interaction of the 3,4-dimethoxy phenyl ring against one of the conserved phenylalanine residues, Phe372 (PDE4D numbering), located in the active site, close to the protein surface (Fig 11). This is a common interaction, that has been observed in other crystal structures of PDE4 inhibitors [33, 34].

In the top pose with PDE4A (Fig 11A), LASSBio-448 performs van der Waals interactions with amino acid residues Ile336, Met337 and Phe340. It makes a π-stacking interaction with Phe372 and makes hydrogen bonds involving one of the oxygen atoms of the sulfonyl group, the hydroxyl of Tyr159 and one of the hydrogen atoms of a water (HOH) molecule, which is part of a network of interactions with Zn^2+^ in the active site of PDE4A.

Analysis of the top pose of LASSBio-448 with PDE4B (Fig 11B) showed that it also performs van der Waals interactions with amino acid residues Ile336, Met337 and Phe340. It makes a close π-stacking interaction with Phe372 and makes a hydrogen bond involving the oxygen atom of the 4-methoxy phenyl group and the NH- group of the conserved Gln369. This docking pose and observed interactions are consistent with other studies with inhibitors that possess the 3,4-dimethoxy phenyl group [34]. LASSBio-448 (**6a**) also performs hydrogen bonds to the -SH group of Cys358 *via* one of the oxygen atoms of the 1,3-benzodioxole ring. Additionally, one of the oxygen atoms of the sulfonyl group is in close contact with the -CH group of His160 and the NE2 nitrogen of His204.

Sulfonamide **6a** (LASSBio-448) makes hydrogen bonds involving the oxygen atoms of the sulfonyl group and the hydroxyl of Tyr159 and a water molecule (HOH) from PDE4C (Fig 11C). It also hydrogen bonds with the conserved Gln369 *via* the 3-methoxy-phenyl oxygens. It showed a π-stacking interaction involving the 3,4-dimethoxy phenyl ring against Phe372 and Van der Waals contacts involving the 3-methoxy-phenyl group and Met337 and the linking hydrocarbon chain and Ile336 (Fig 11C).

Finally, the top pose of LASSBio-448 and PDE4D (Fig 11D) showed that it is capable of performing very similar interactions compared to the ones with PDE4B. The only exception is that, instead of being in close contact with the NE2 nitrogen of His204 it hydrogen bonds to HOH1006, which is part of the network of interactions involving Mg^2+^ in the active site.

**Fig 12:**
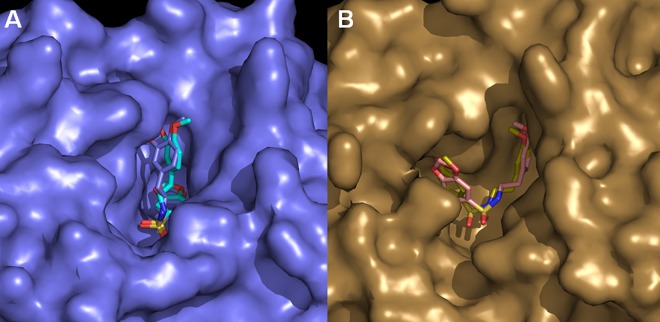
Superimposition of the top poses of LASSBio-448 obtained by docking with PDE4A and PDE4C (light purple surface, PDE4A) (A), PDE4B and PDE4D (gold surface, PDE4D) (B). Hydrogen atoms have been omitted for clarity.

With both PDE4A and PDE4C the theoretical binding modes are very similar. The 3,4-dimethoxy phenyl ring of LASSBio-448 (**6a**) interacts closer to the conserved Gln369 at the solvent accessible surface (Fig 12A) in a folded conformation which does not span the whole active site and in which the 1,3-benzodioxole ring is buried within the pocket. With PDE4B and PDE4D, we have also observed similar conformations (Fig 12B). However, with these isoforms, LASSBio-448 interacts in a more extended conformation in which the 3,4-dimethoxy phenyl and the 1,3-benzodioxole rings are closer to the protein surface and fit the pocket making more contacts. Maybe this is responsible for the similar IC_50_ values observed for LASSBio-448 (**6a**) against these recombinant isoforms (Table 2). It is worth of note that the active sites of PDE4B and PDE4D are considered comparable [33], while PDE4A has shown displacements of aminoacid residues close to the conserved Glutamine (Gln369). Interestingly, our PDE4C model was based on a PDE4D template but resembles more PDE4A. Overall, different binding conformations and observed affinities may be due to subtle but important differences amongst the active sites of the studied PDE4, especially PDE4A, which has been reported to present conformational divergences compared to PDE4B and PDE4D [34].

## Conclusion

In summary, we identified a new PDE4 inhibitor, equipotent to the standard rolipram. This inhibitor (**6a**) has been shown to be orally active in murine model of asthma. LASSBio-448 (**6a**) prevented the up-regulation of eosinophil levels in bronchoalveolar lavage fluid, impaired the progress of allergen-induced eosinophil accumulation in the lung tissue, reduced mucus and extracellular matrix production, was effective in the inhibition of airway hyper-reactivity to methacholine and reduced the levels of IL-4, IL-5, IL-13 and eotaxin-2. Moreover, it was at least 7-fold less pro-emetic than rolipram. Its liver microsomal metabolism was recently described [35]. Docking analysis of the putative interactions of LASSBio-448 (**6a**) with binding site of human PDE4 isoenzymes was performed and will be used to guide the further optimization of this new antiasthmatic lead-candidate.

## Supporting Information

S1 Fig Inhibition of carbachol-stimulated contraction of guinea-pig trachea in vitro by LASSBio-448 is related to increased intracellular cAMP levels in airway smooth muscle cells. (A) Effect of LASSBio-448 on tracheal contraction induced by carbachol (10–8–10–4 M). The data are expressed as the percentage of contractile responses induced by 2.5 μM carbachol. Each value represents the mean ± SEM from at least 4 animals. (B) Effect of LASSBio-448 treatment on guinea pig tracheal smooth muscle cells cAMP intracellular levels. Cells were treated for 20 min with either LASSBio-448 or forskolin in the presence or absence of SQ22536. Control group received an equal amount of vehicle (DMSO 0.1%). Values represent the mean ± SEM from at least 3 animals. + P<0.05 as compared to vehicle-treated group; *P<0.05 as compared to LASSBio-448-treated group; #P<0.05 as compared to forskolin-treated group.
(TIFF)

S2 Fig Representative histological changes noted 24 h after the series of three ovalbumin challenges, done at days 14, 21 and 28 post-sensitization. (a) Photomicrograph of paraffin-embedded lung section stained by hematoxilin-eosin indicating peribronchial inflammatory infiltrate, (b) Photomicrograph taken of representative airways showing goblet-cell hyperplasia and mucus production (purple color, arrowheads), and (c) Photomicrograph of representative lung histologic section stained with Gomori trichrome revealing peribronchial fibrosis. Original magnifications of x400.
(TIFF)

S3 Fig ^13^C NMR spectrum of 6a (50 MHz, CDCl3). (TIF)

S4 Fig ^1^H NMR spectrum of 6a (200 MHz, CDCl3). (TIF)

S5 Fig ^13^C NMR spectrum of 6f (125 MHz, CDCl3). (TIF)

S6 Fig ^1^H NMR spectrum of 6f (500 MHz, CDCl3). (TIF)

S7 Fig ^13^C NMR spectrum of 6j (125 MHz, CDCl3). (TIF)

S8 Fig ^1^H NMR spectrum of 6j (500 MHz, CDCl3). (TIF)

S9 Fig ^13^C NMR spectrum of 6b (50 MHz, CDCl3). (TIF)

S10 Fig ^1^H NMR spectrum of 6b (200 MHz, CDCl3). (TIF)

S11 Fig ^13^C NMR spectrum of 6c (50 MHz, CDCl3). (TIF)

S12 Fig ^1^H NMR spectrum of 6c (200 MHz, CDCl3). (TIF)

S13 Fig ^13^C NMR spectrum of 6d (125 MHz, CDCl3). (TIF)

S14 Fig ^1^H NMR spectrum of 6d (500 MHz, CDCl3). (TIF)

S15 Fig ^1^H NMR spectrum of 6e (200 MHz, CDCl3). (TIF)

S16 Fig ^13^C NMR spectrum of 6g (50 MHz, CDCl3). (TIF)

S17 Fig ^1^H NMR spectrum of 6g (200 MHz, CDCl3). (TIF)

S18 Fig ^13^C NMR spectrum of 6i (125 MHz, CDCl3). (TIF)

S19 Fig ^1^H NMR spectrum of 6i (500 MHz, CDCl3). (TIF)

S20 Fig ^13^C NMR spectrum of 6h (50 MHz, CDCl3). (TIF)

S21 Fig ^1^H NMR spectrum of 6h (200 MHz, CDCl3). (TIF)

S22 Fig ^13^C NMR spectrum of 6k (125 MHz, CDCl3). (TIF)

S23 Fig ^1^H NMR spectrum of 6k (200 MHz, CDCl3). (TIF)
