# Supplementary figures and images for: Synthesis, Pharmacological Profile and Docking Studies of New Sulfonamides Designed as Phosphodiesterase-4 Inhibitors
Source: PLoS One. 2016 Oct 3;11(10):e0162895. doi: 10.1371/journal.pone.0162895 (PMC5047629; doi:10.1371/journal.pone.0162895)

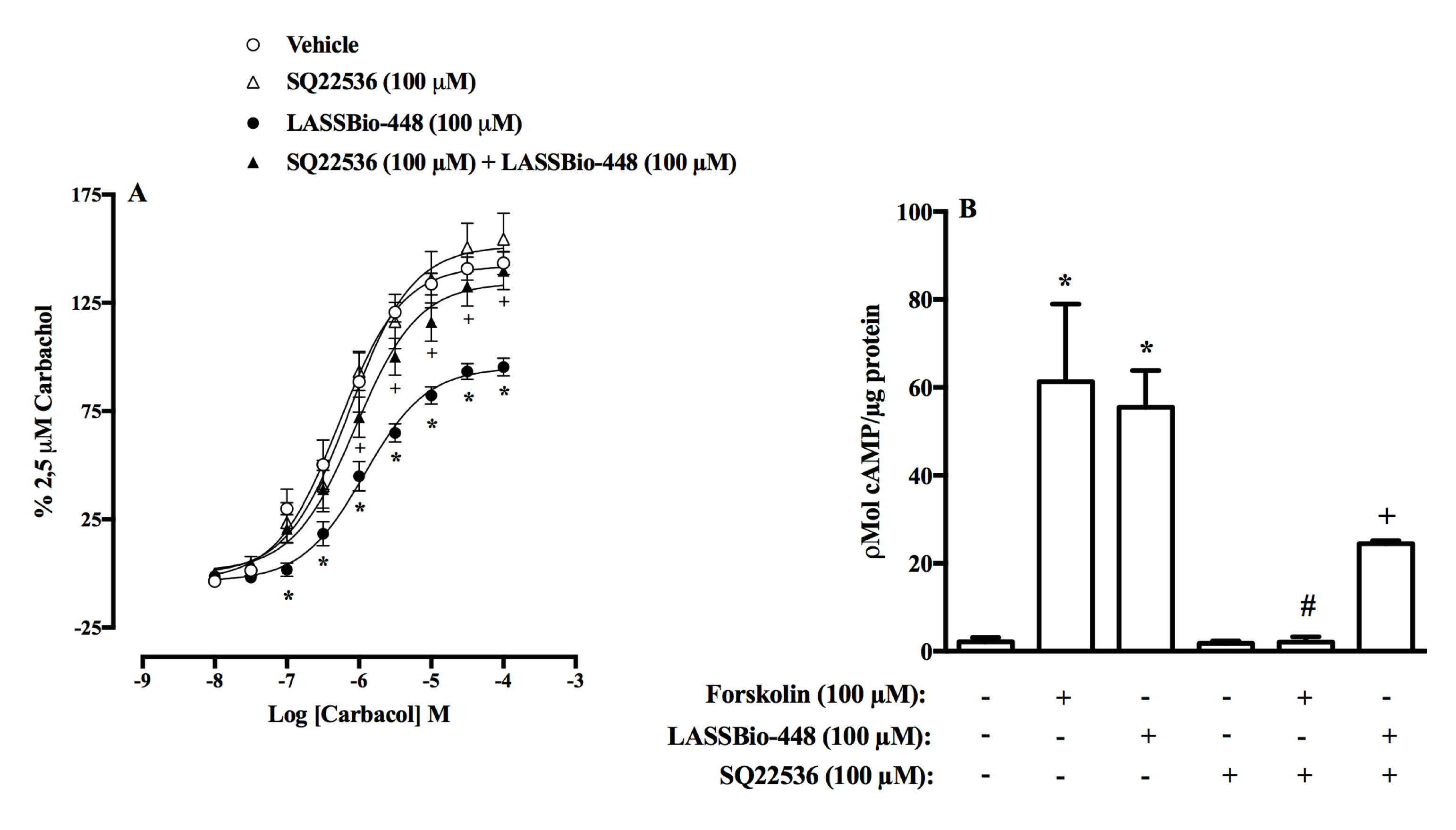

Supplement: S1 Fig — (A) Effect of LASSBio-448 on tracheal contraction induced by carbachol (10–8–10–4 M). The data are expressed as the percentage of contractile responses induced by 2.5 μM carbachol. Each value represents the mean ± SEM from at least 4 animals. (B) Effect of LASSBio-448 treatment on guinea pig tracheal smooth muscle cells cAMP intracellular levels. Cells were treated for 20 min with either LASSBio-448 or forskolin in the presence or absence of SQ22536. Control group received an equal amount of vehicle (DMSO 0.1%). Values represent the mean ± SEM from at least 3 animals. + P<0.05 as compared to vehicle-treated group; *P<0.05 as compared to LASSBio-448-treated group; #P<0.05 as compared to forskolin-treated group. (TIFF) [file pone.0162895.s001.tiff]

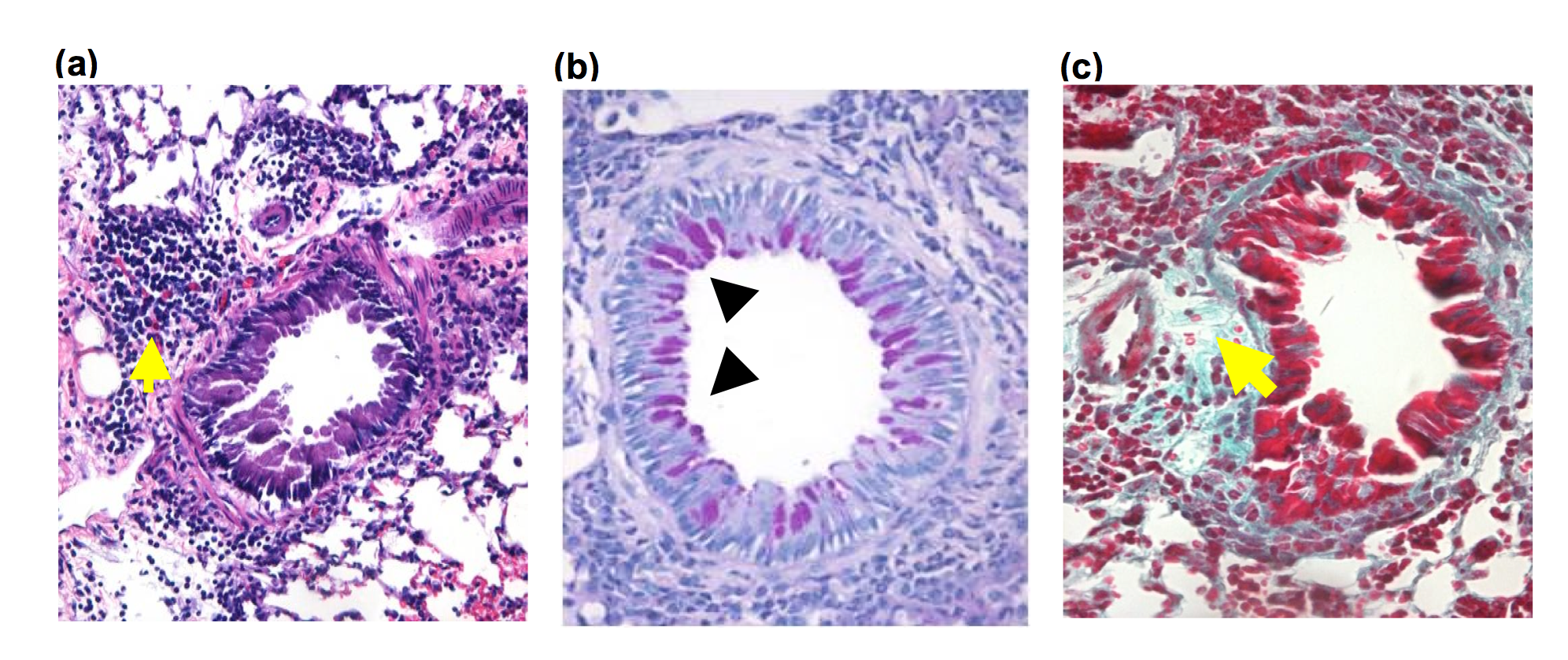

Supplement: S2 Fig — (a) Photomicrograph of paraffin-embedded lung section stained by hematoxilin-eosin indicating peribronchial inflammatory infiltrate, (b) Photomicrograph taken of representative airways showing goblet-cell hyperplasia and mucus production (purple color, arrowheads), and (c) Photomicrograph of representative lung histologic section stained with Gomori trichrome revealing peribronchial fibrosis. Original magnifications of x400. (TIFF) [file pone.0162895.s002.tiff]

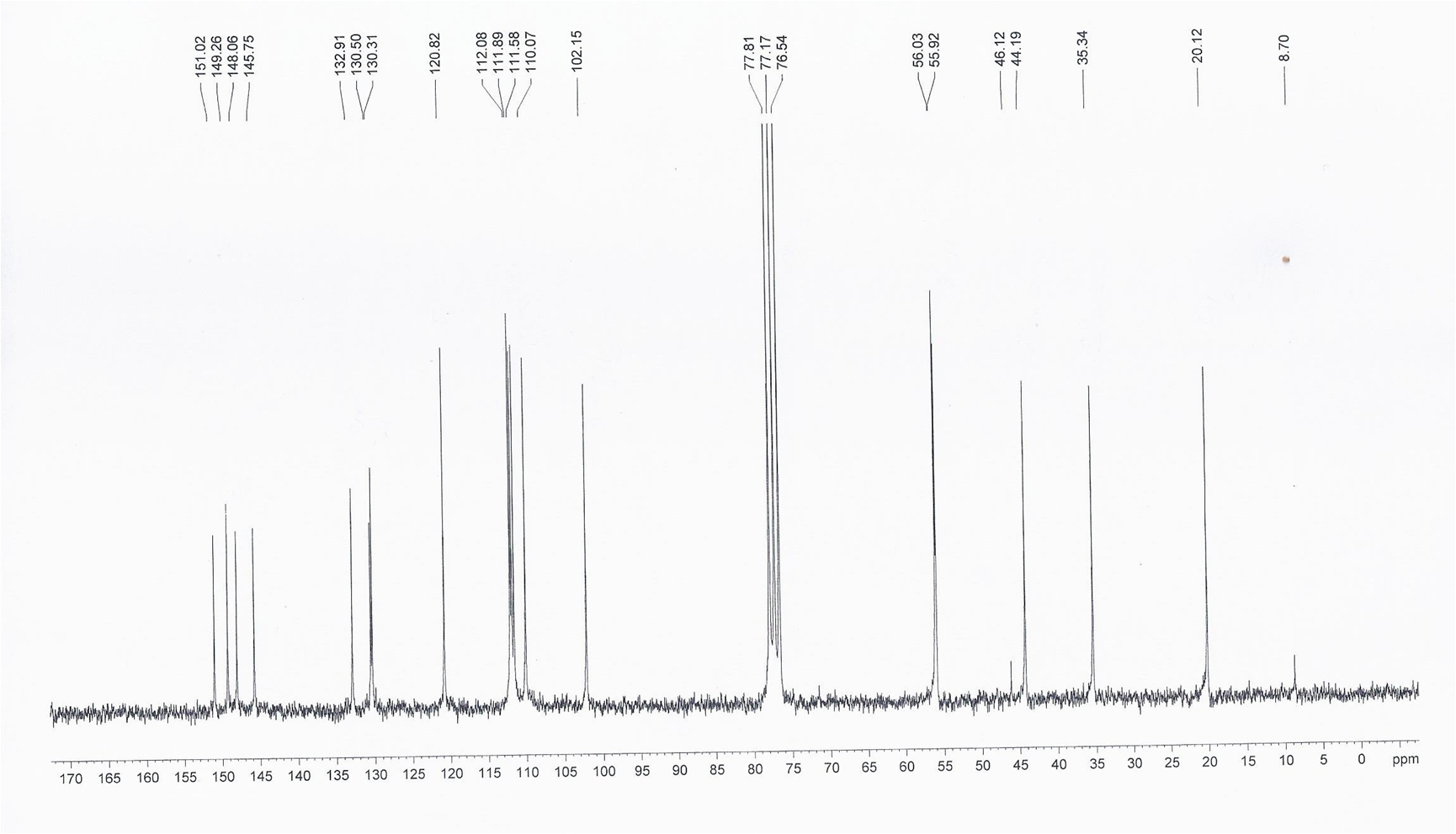

Supplement: S3 Fig — (TIF) [file pone.0162895.s003.tif]

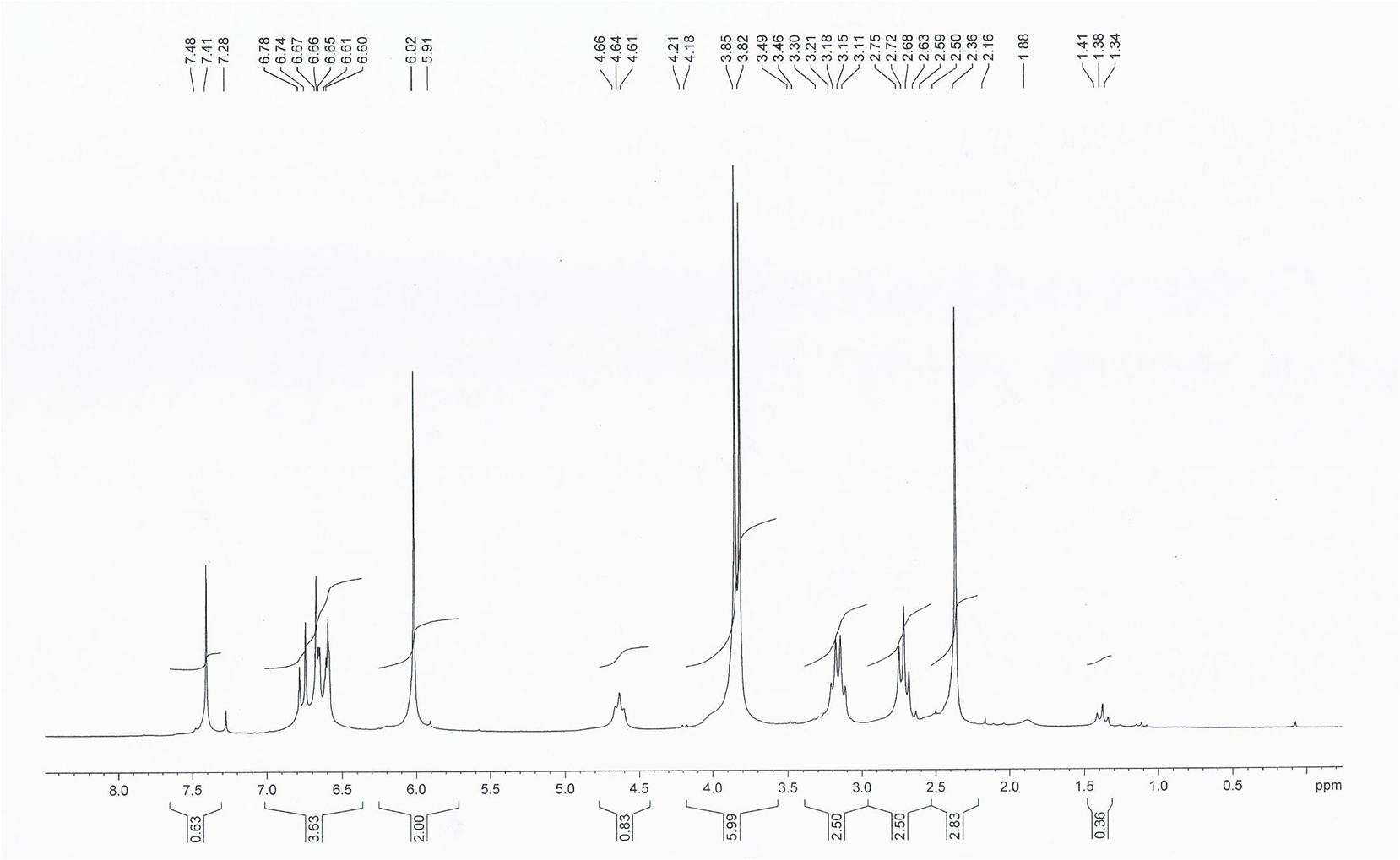

Supplement: S4 Fig — (TIF) [file pone.0162895.s004.tif]

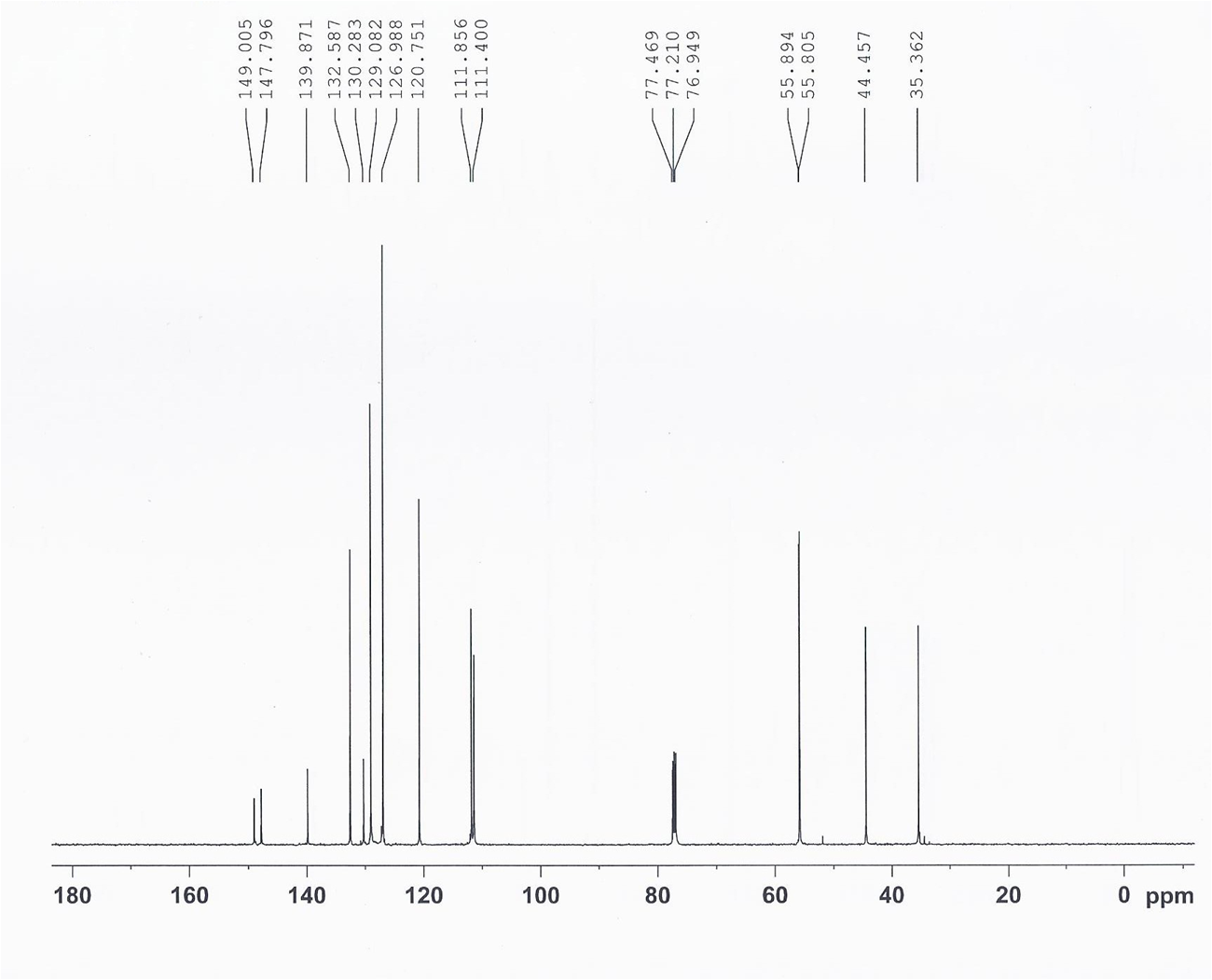

Supplement: S5 Fig — (TIF) [file pone.0162895.s005.tif]

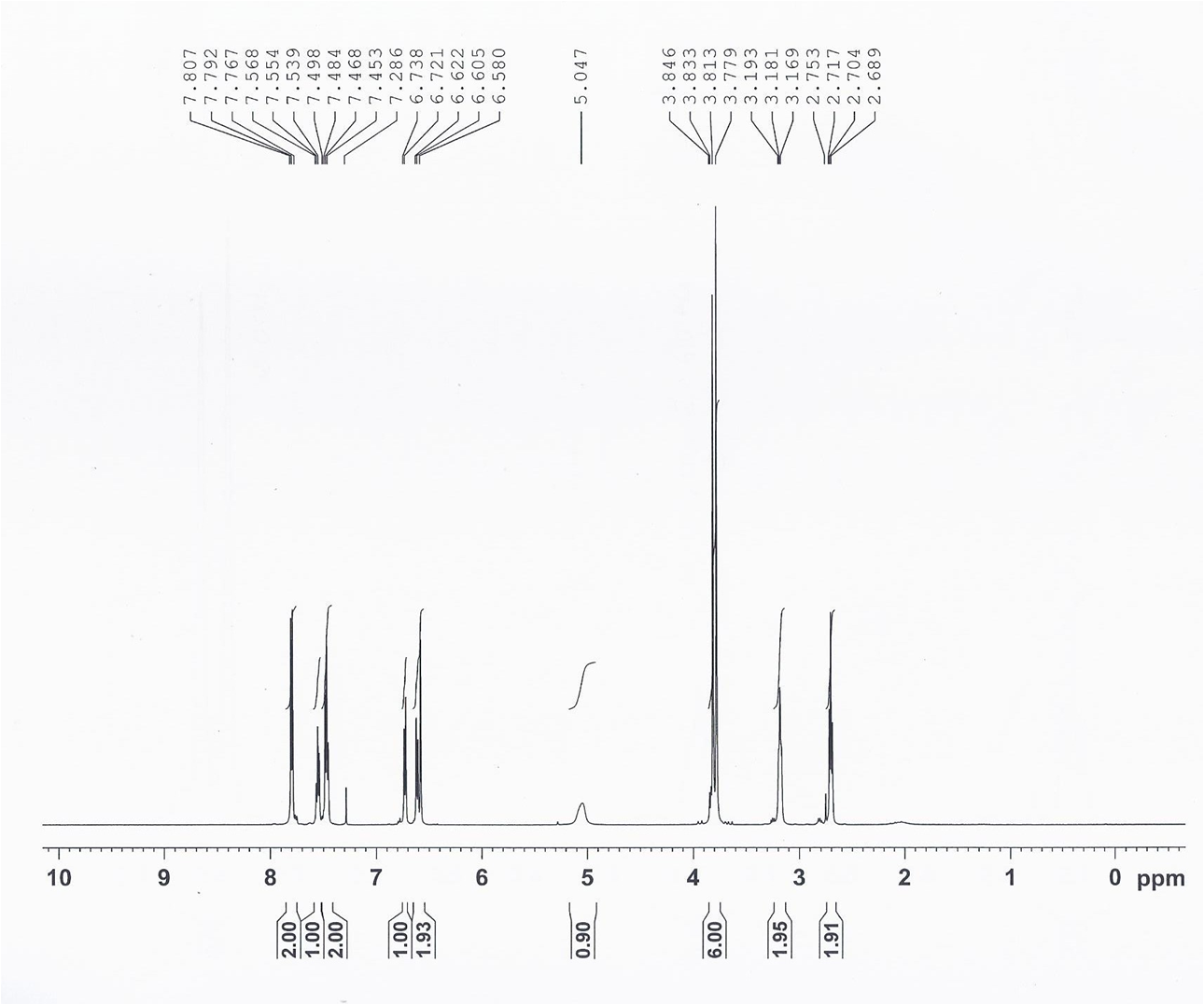

Supplement: S6 Fig — (TIF) [file pone.0162895.s006.tif]

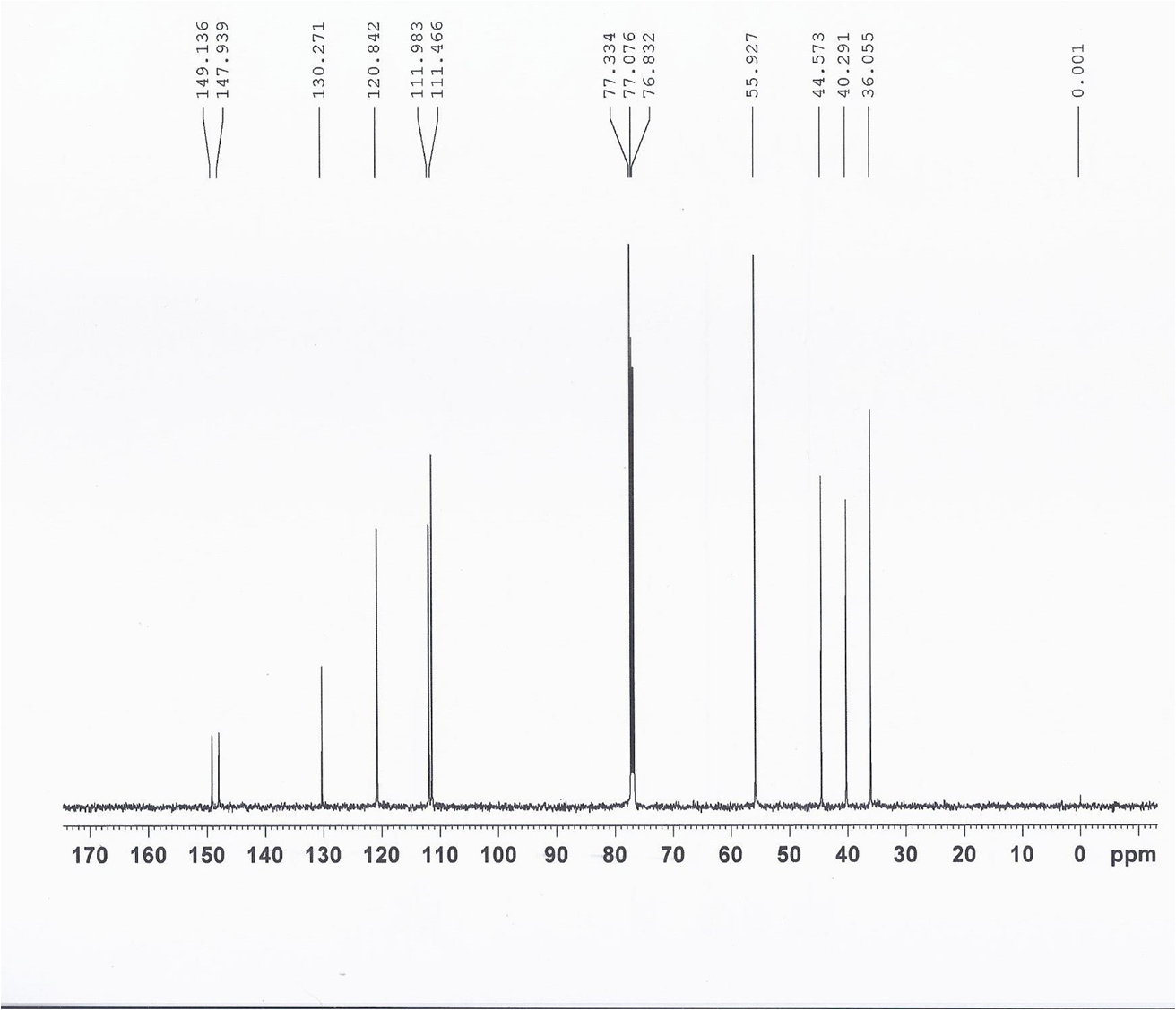

Supplement: S7 Fig — (TIF) [file pone.0162895.s007.tif]

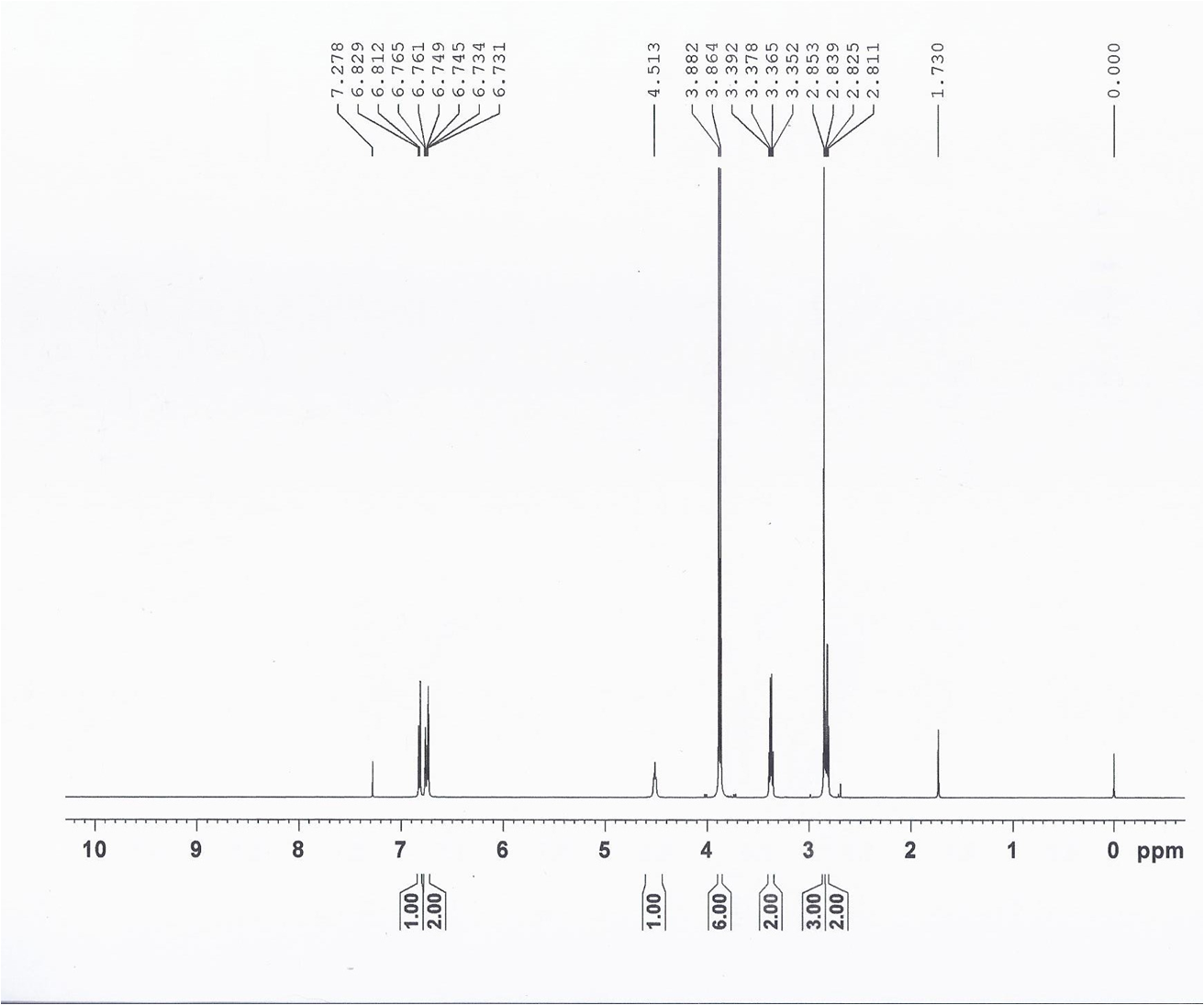

Supplement: S8 Fig — (TIF) [file pone.0162895.s008.tif]

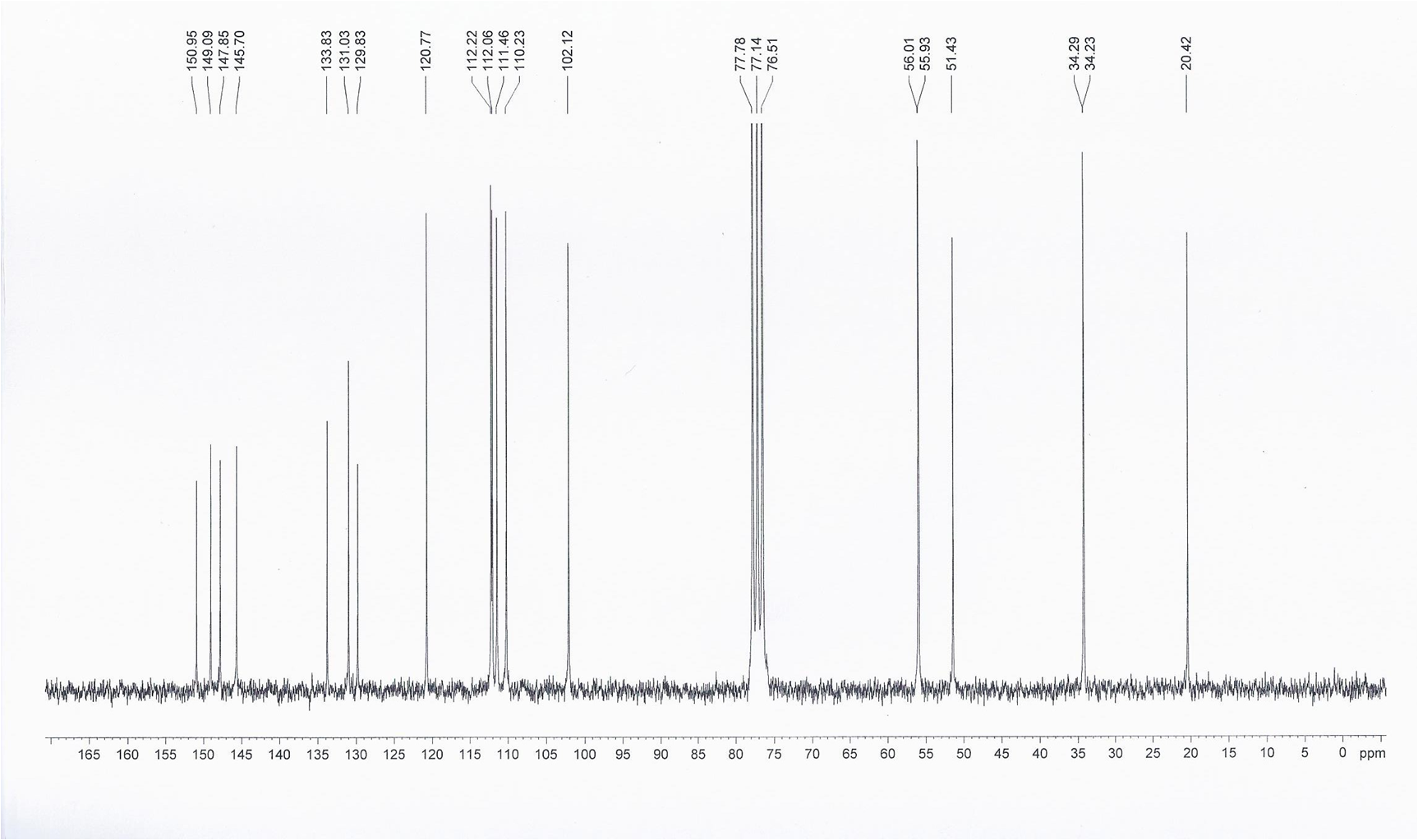

Supplement: S9 Fig — (TIF) [file pone.0162895.s009.tif]

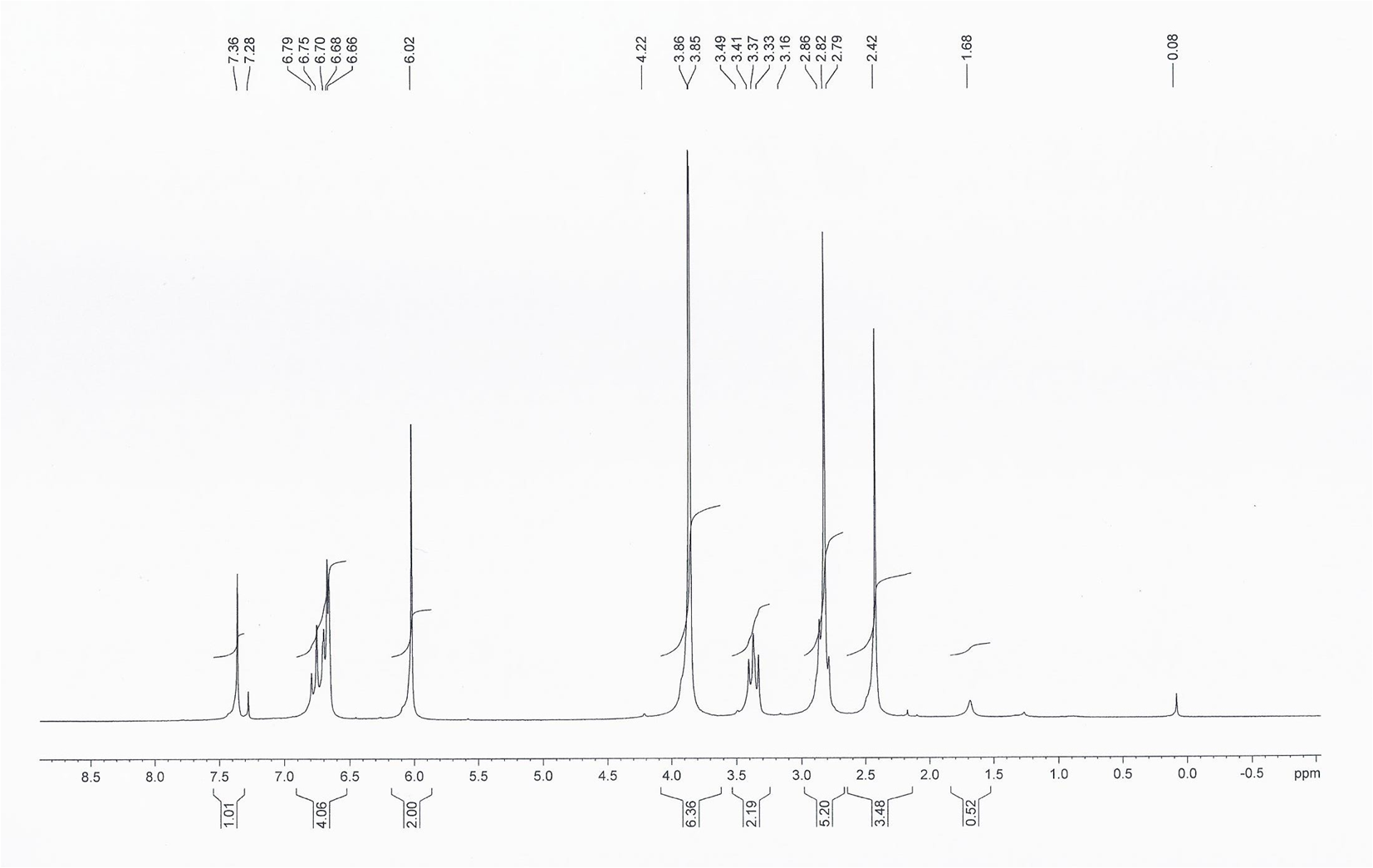

Supplement: S10 Fig — (TIF) [file pone.0162895.s010.tif]

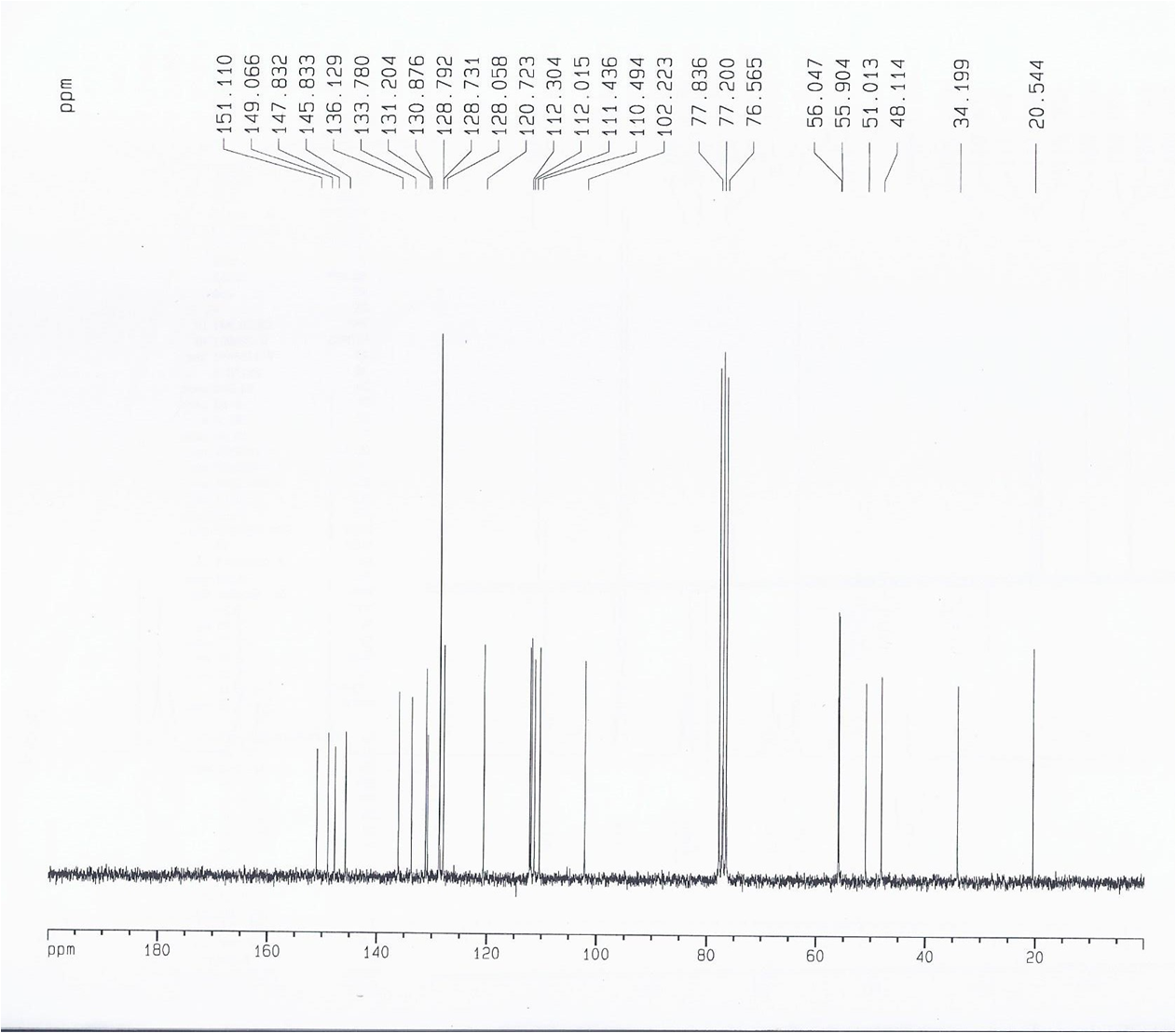

Supplement: S11 Fig — (TIF) [file pone.0162895.s011.tif]

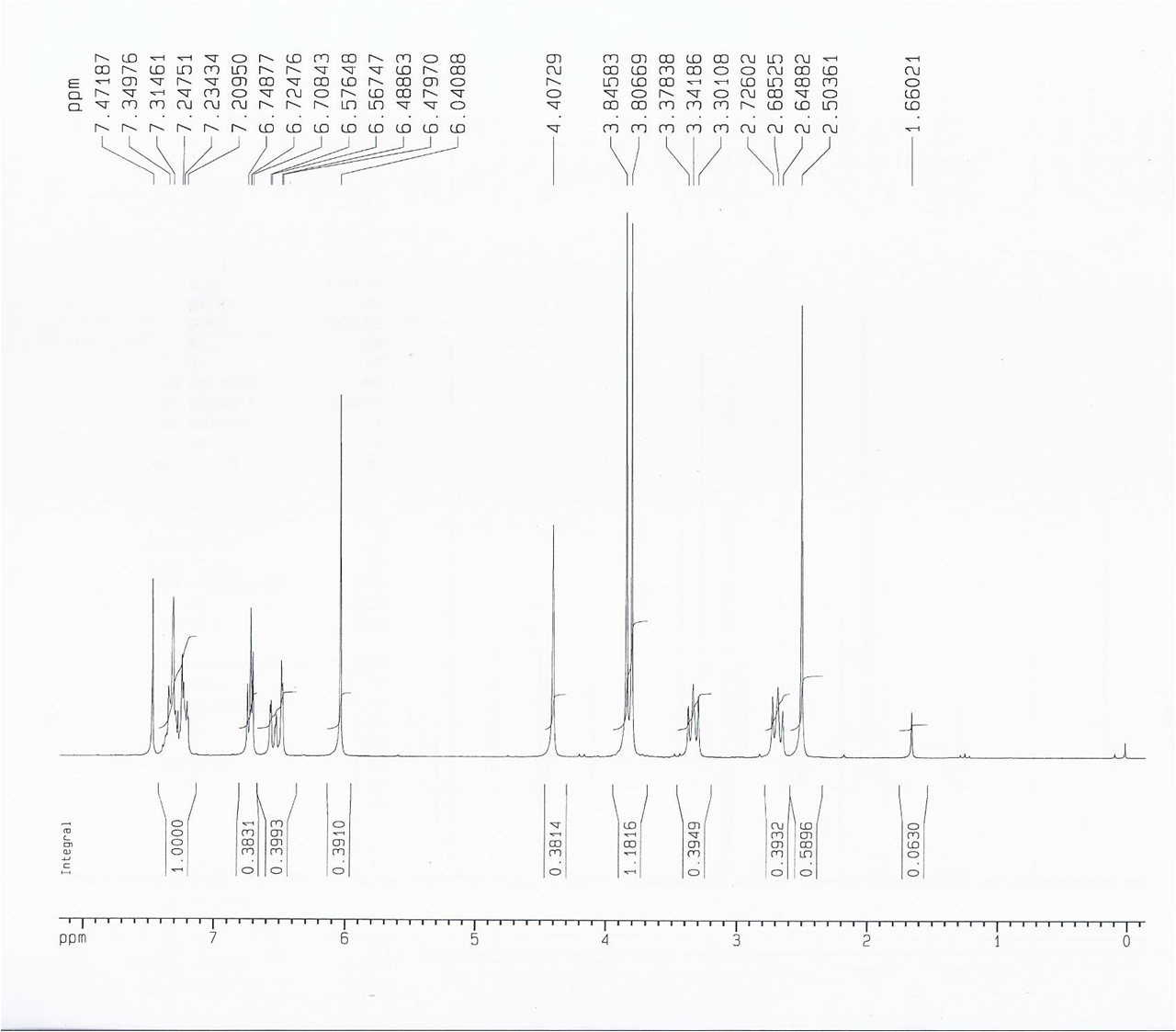

Supplement: S12 Fig — (TIF) [file pone.0162895.s012.tif]

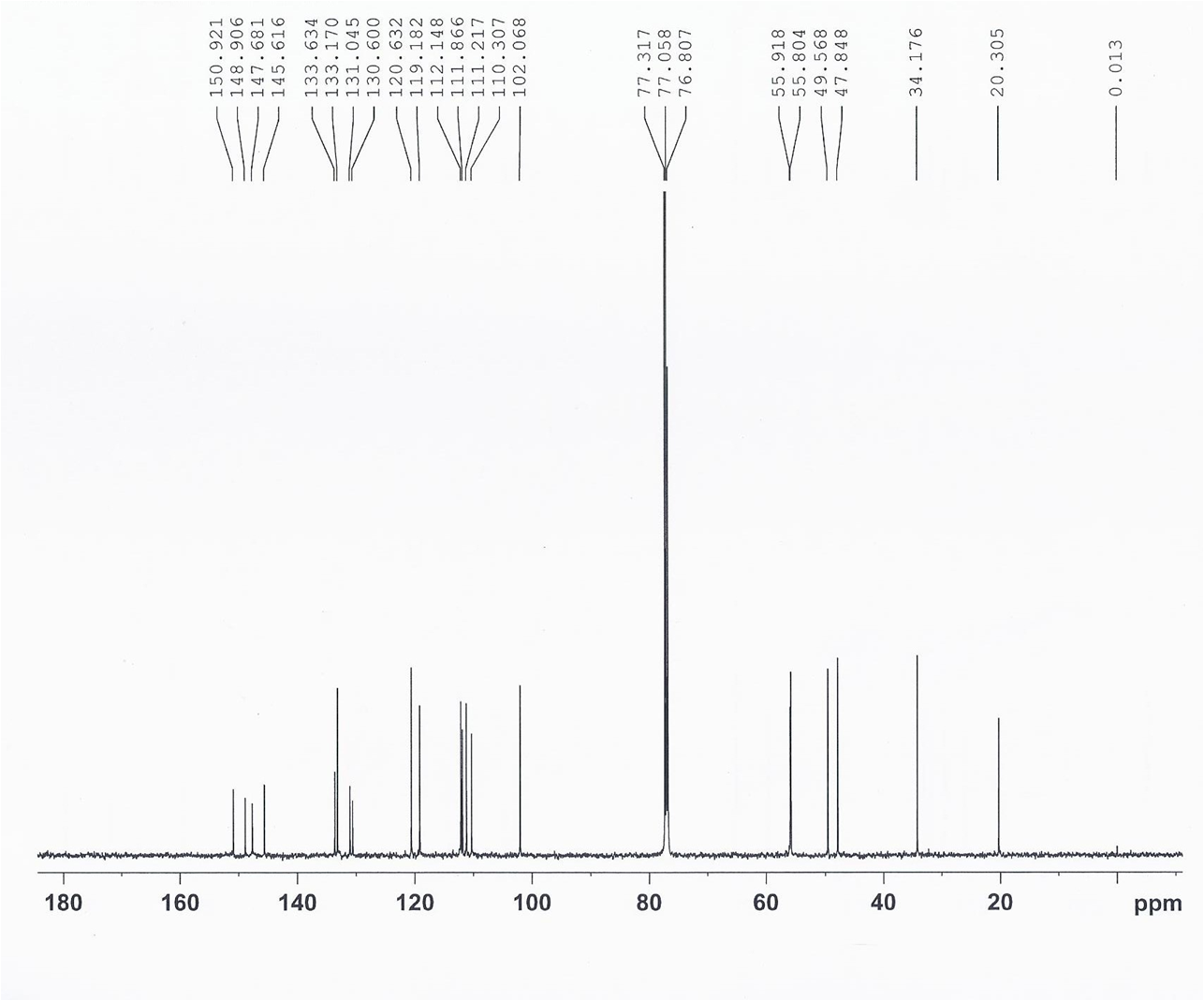

Supplement: S13 Fig — (TIF) [file pone.0162895.s013.tif]

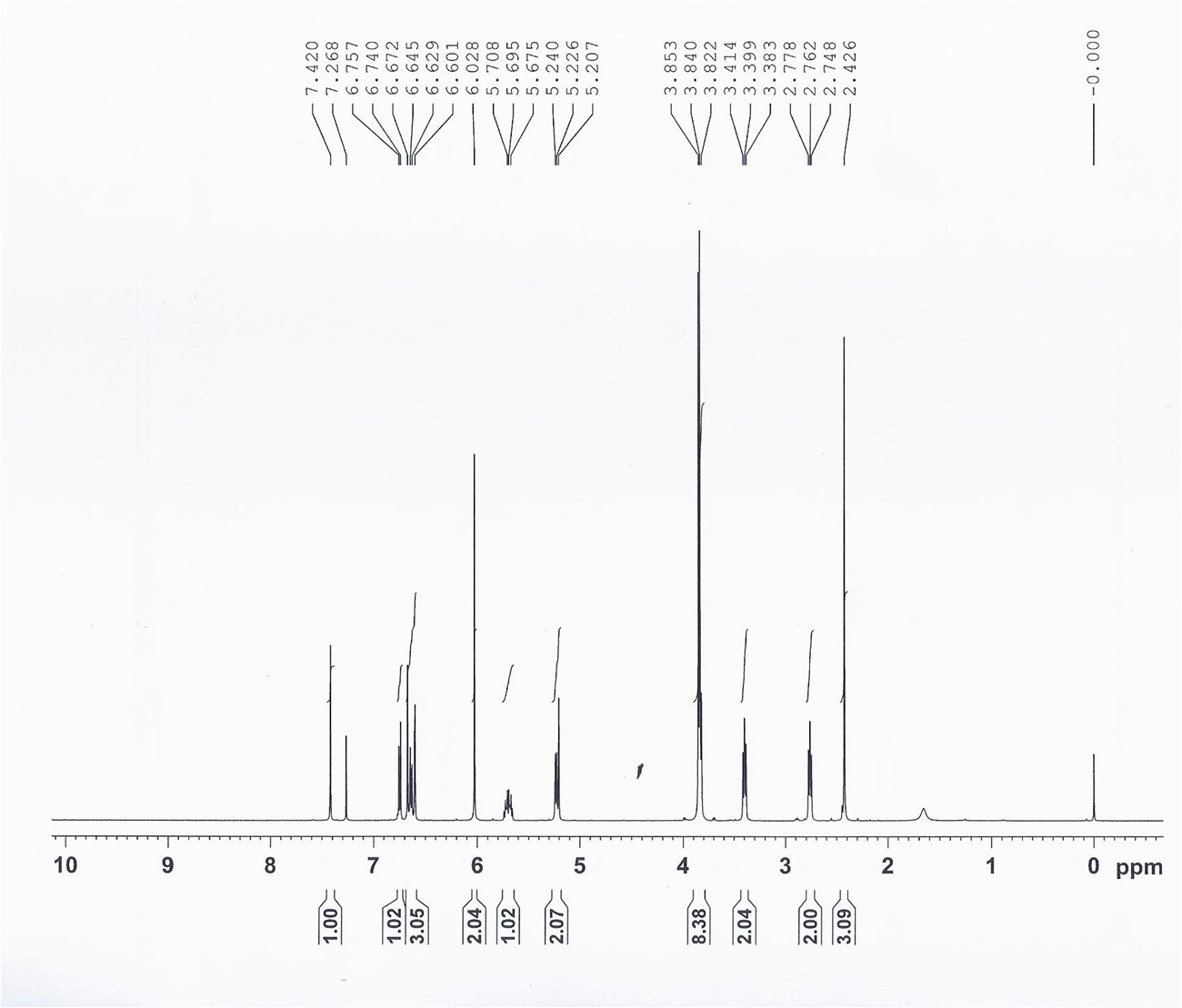

Supplement: S14 Fig — (TIF) [file pone.0162895.s014.tif]

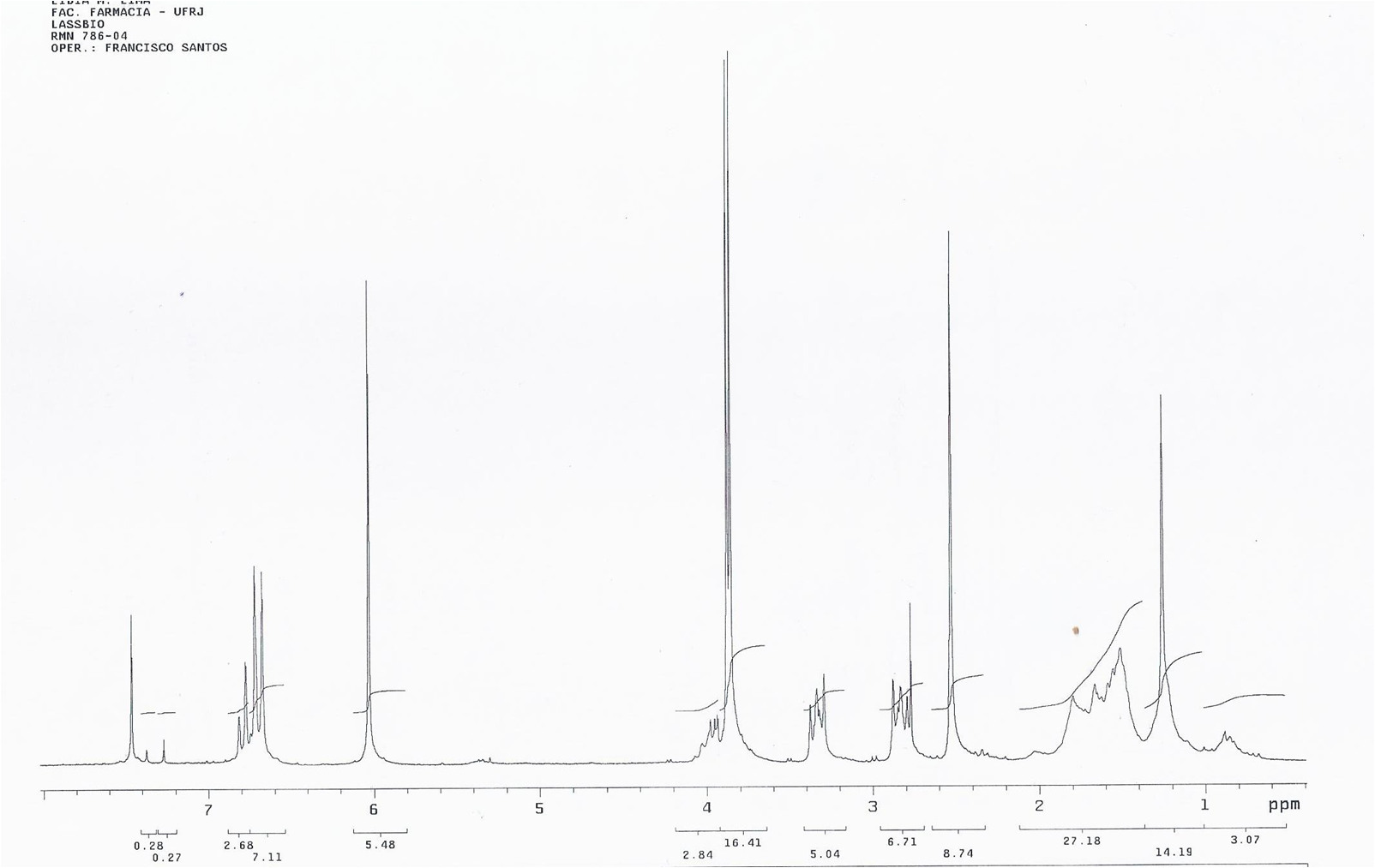

Supplement: S15 Fig — (TIF) [file pone.0162895.s015.tif]

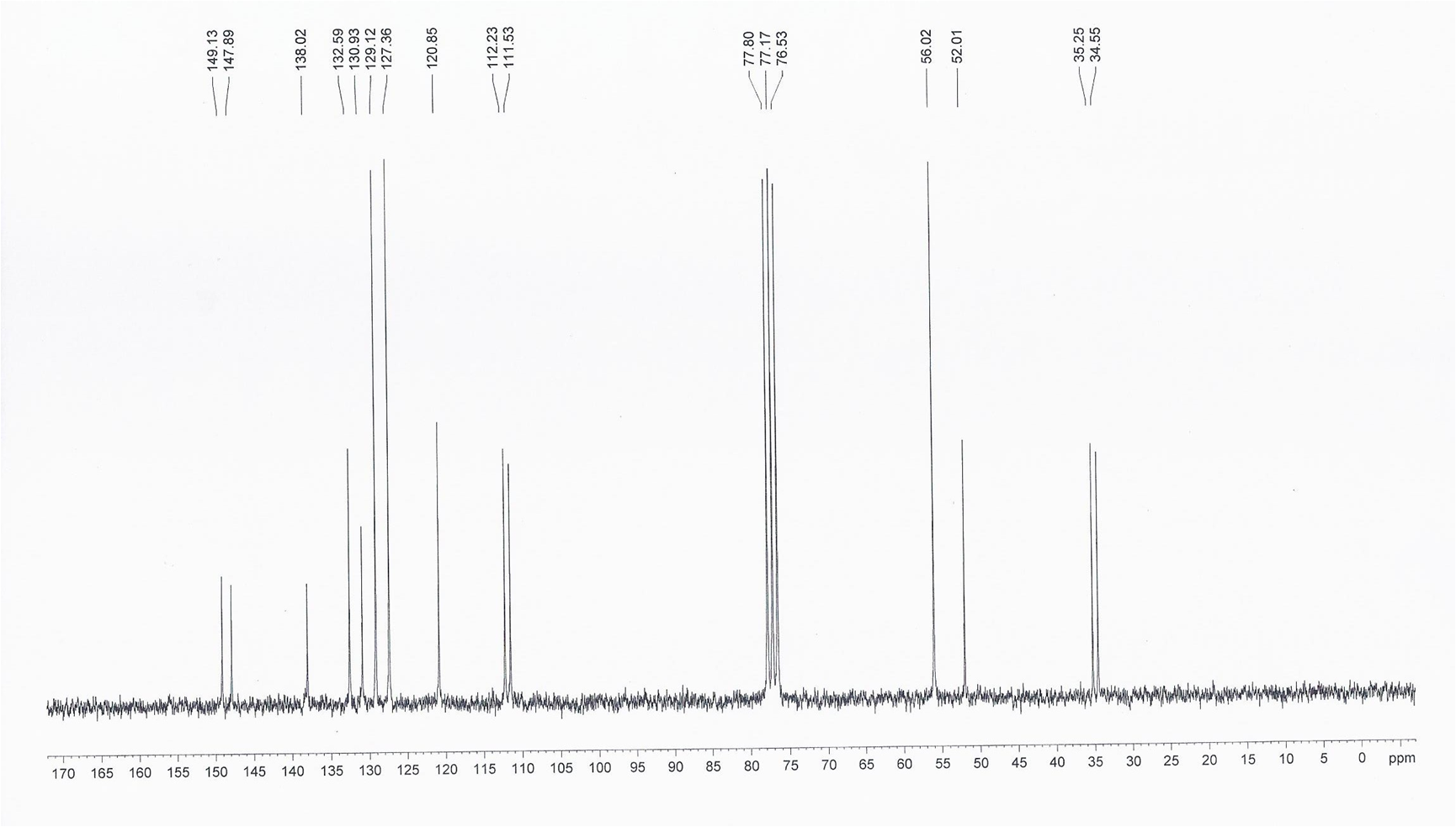

Supplement: S16 Fig — (TIF) [file pone.0162895.s016.tif]

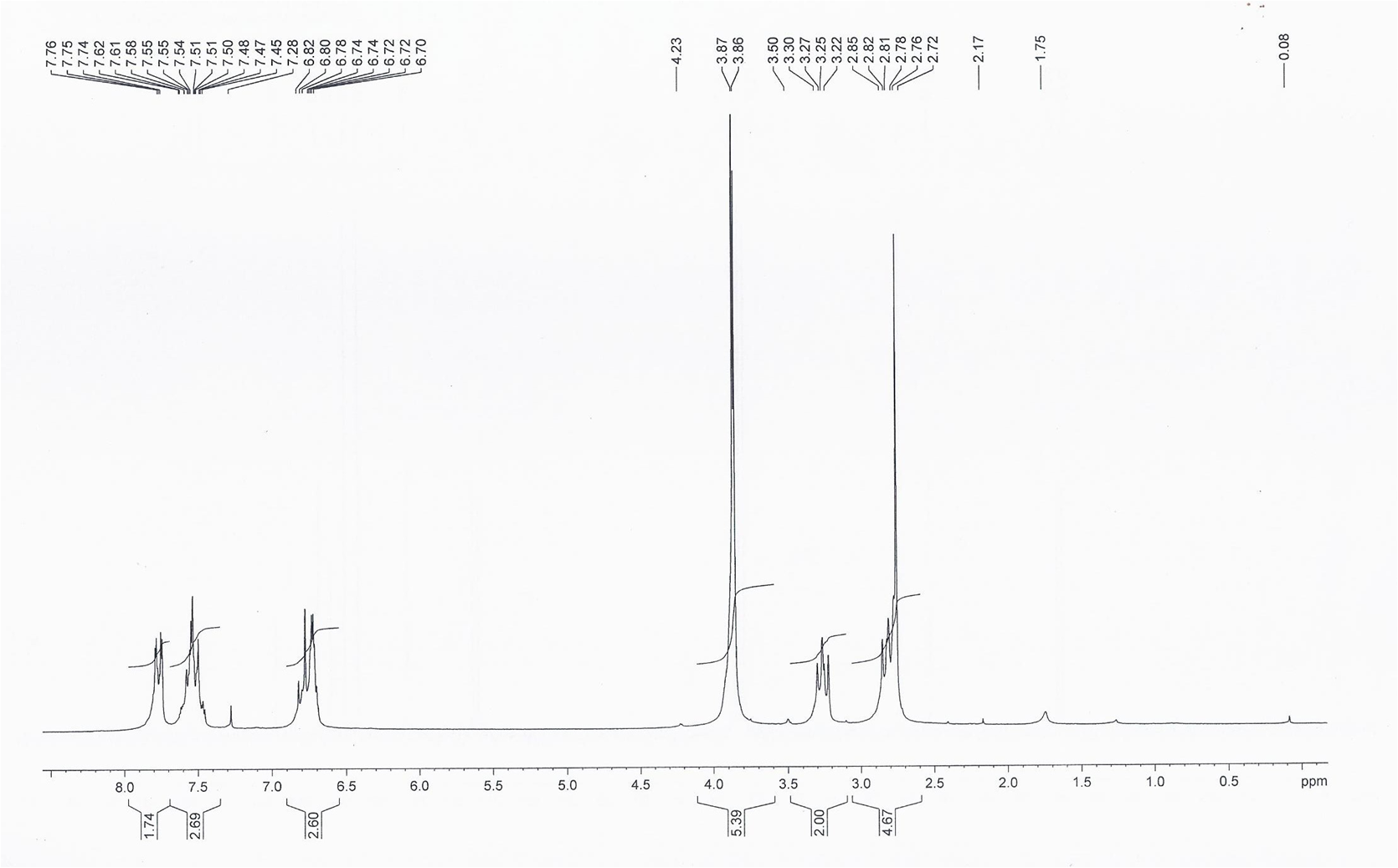

Supplement: S17 Fig — (TIF) [file pone.0162895.s017.tif]

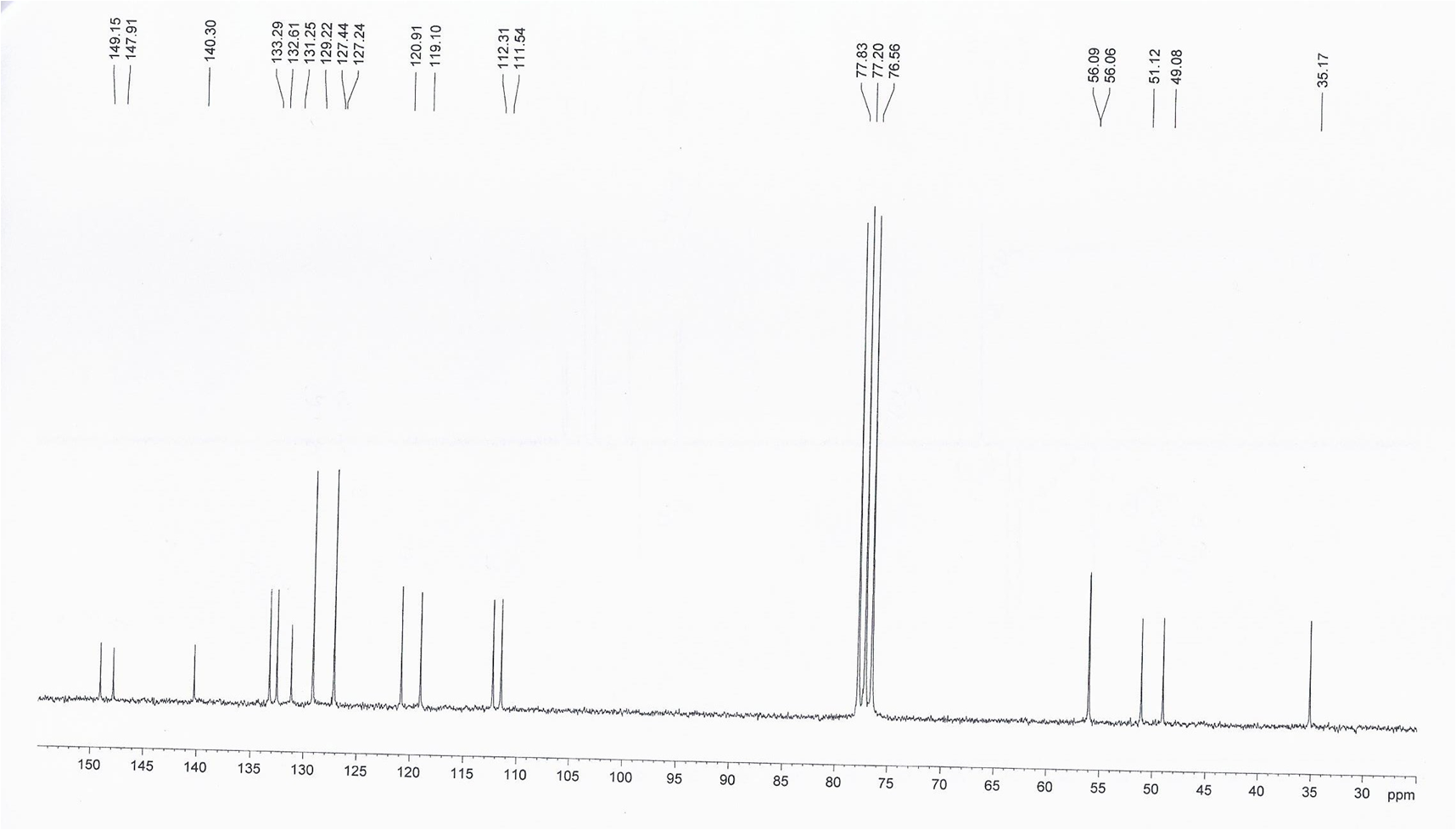

Supplement: S18 Fig — (TIF) [file pone.0162895.s018.tif]

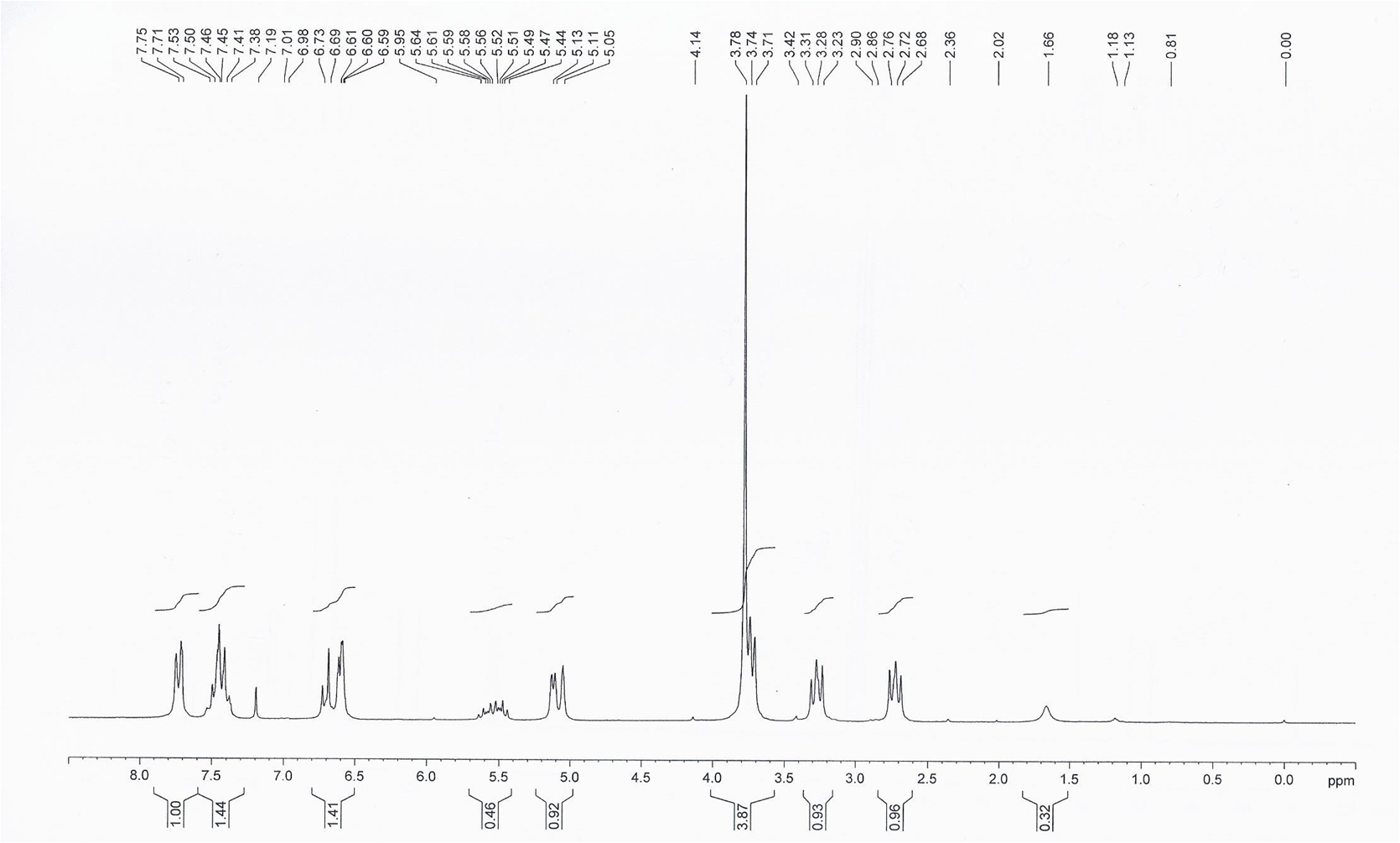

Supplement: S19 Fig — (TIF) [file pone.0162895.s019.tif]

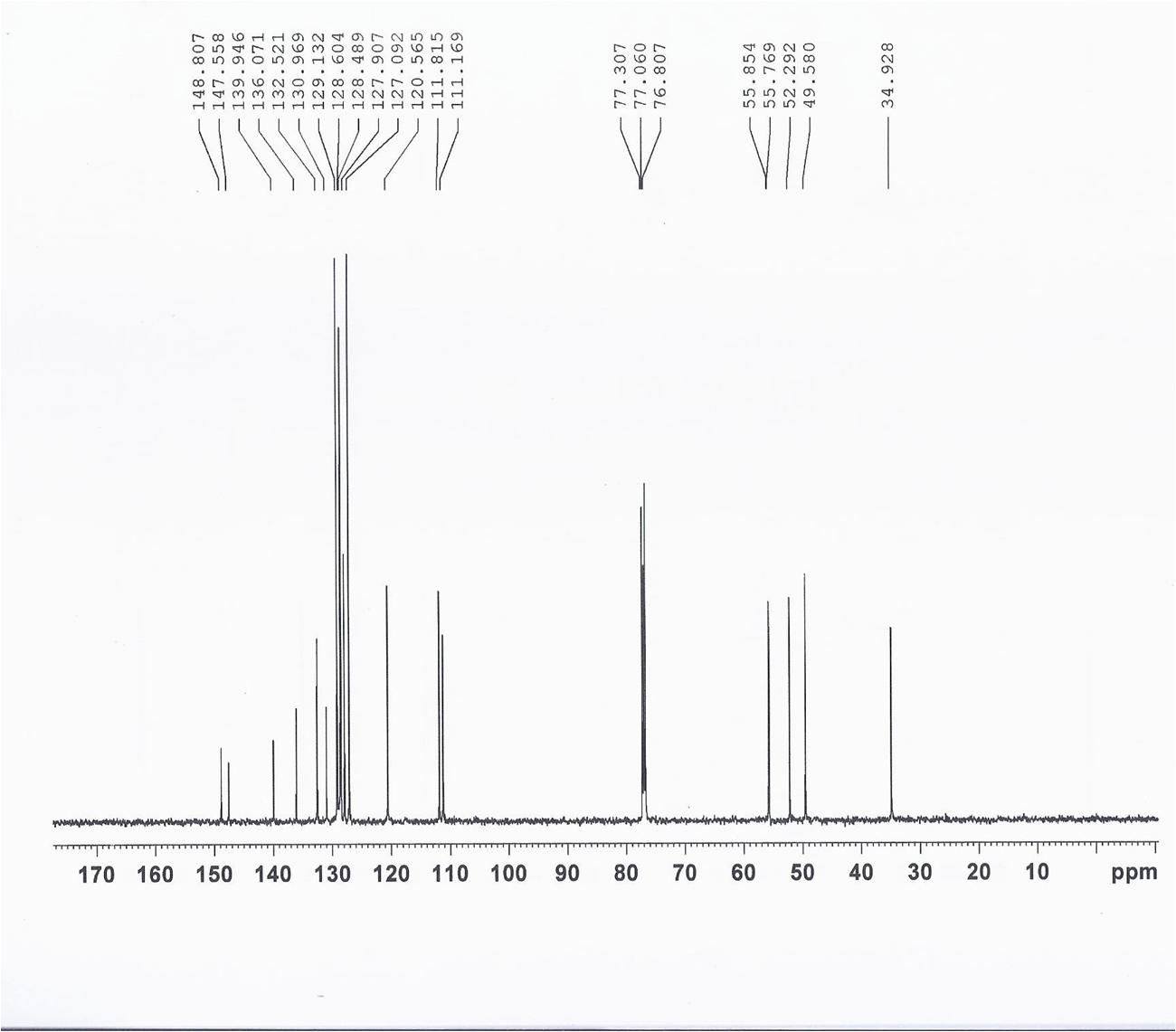

Supplement: S20 Fig — (TIF) [file pone.0162895.s020.tif]

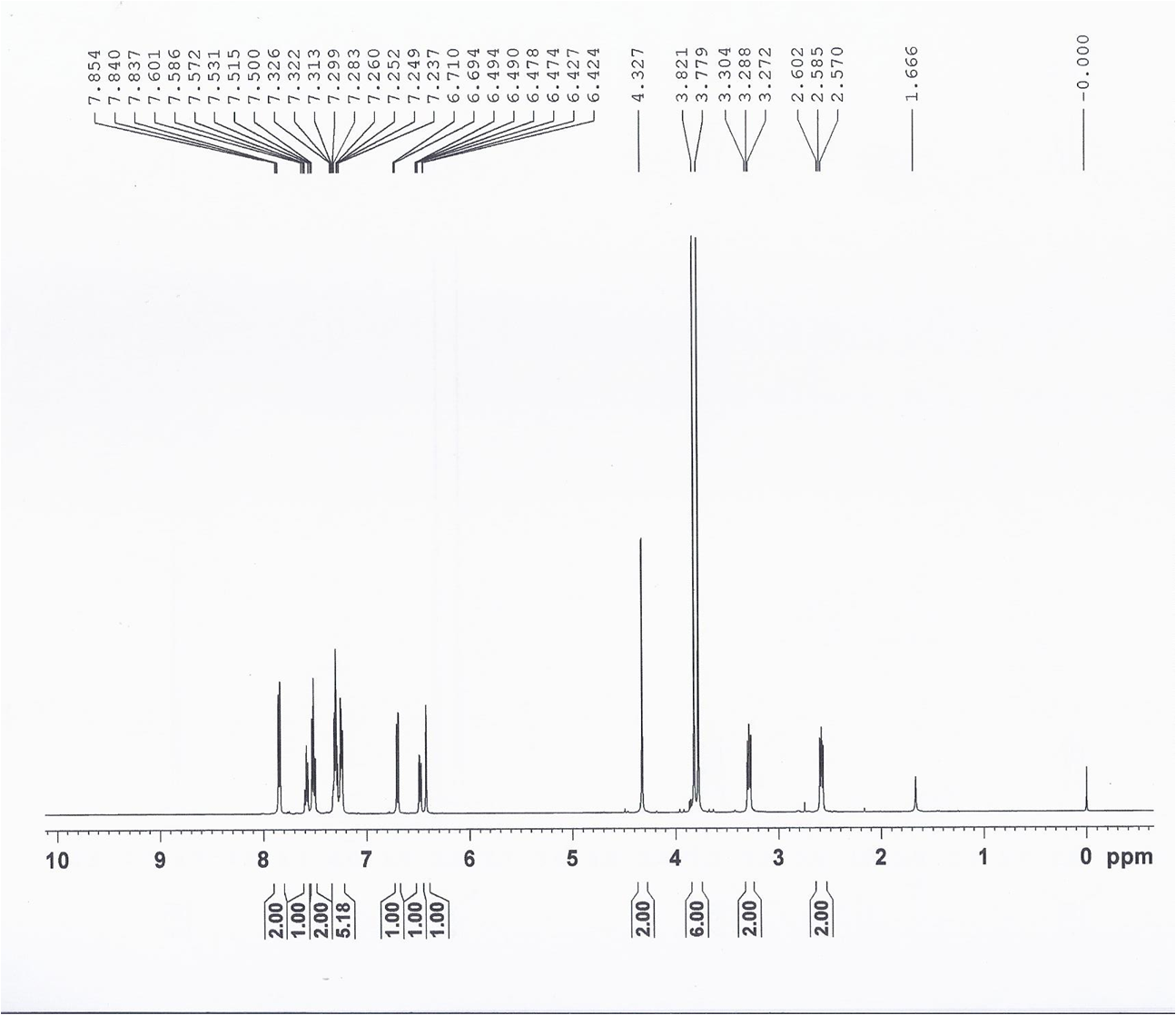

Supplement: S21 Fig — (TIF) [file pone.0162895.s021.tif]

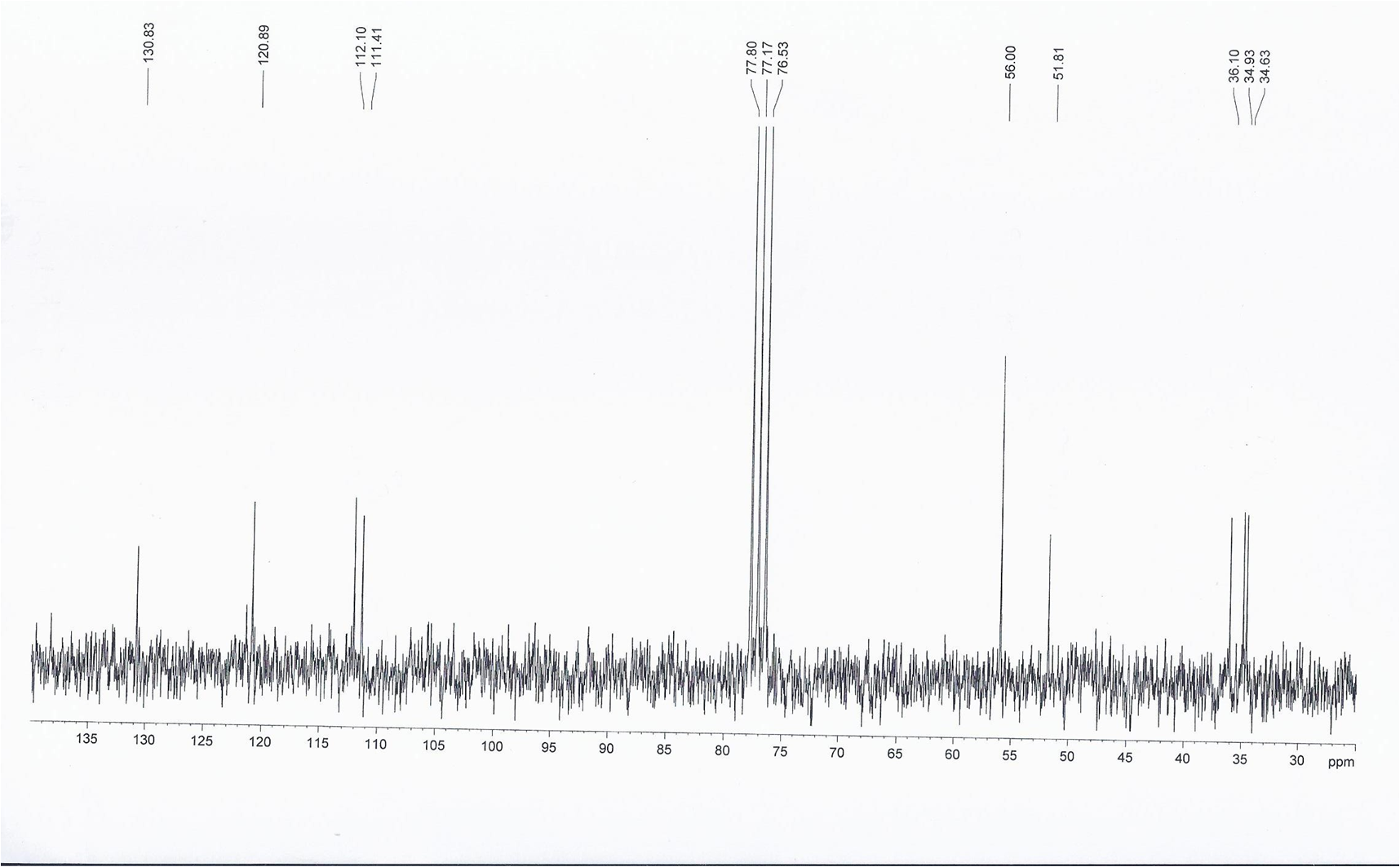

Supplement: S22 Fig — (TIF) [file pone.0162895.s022.tif]

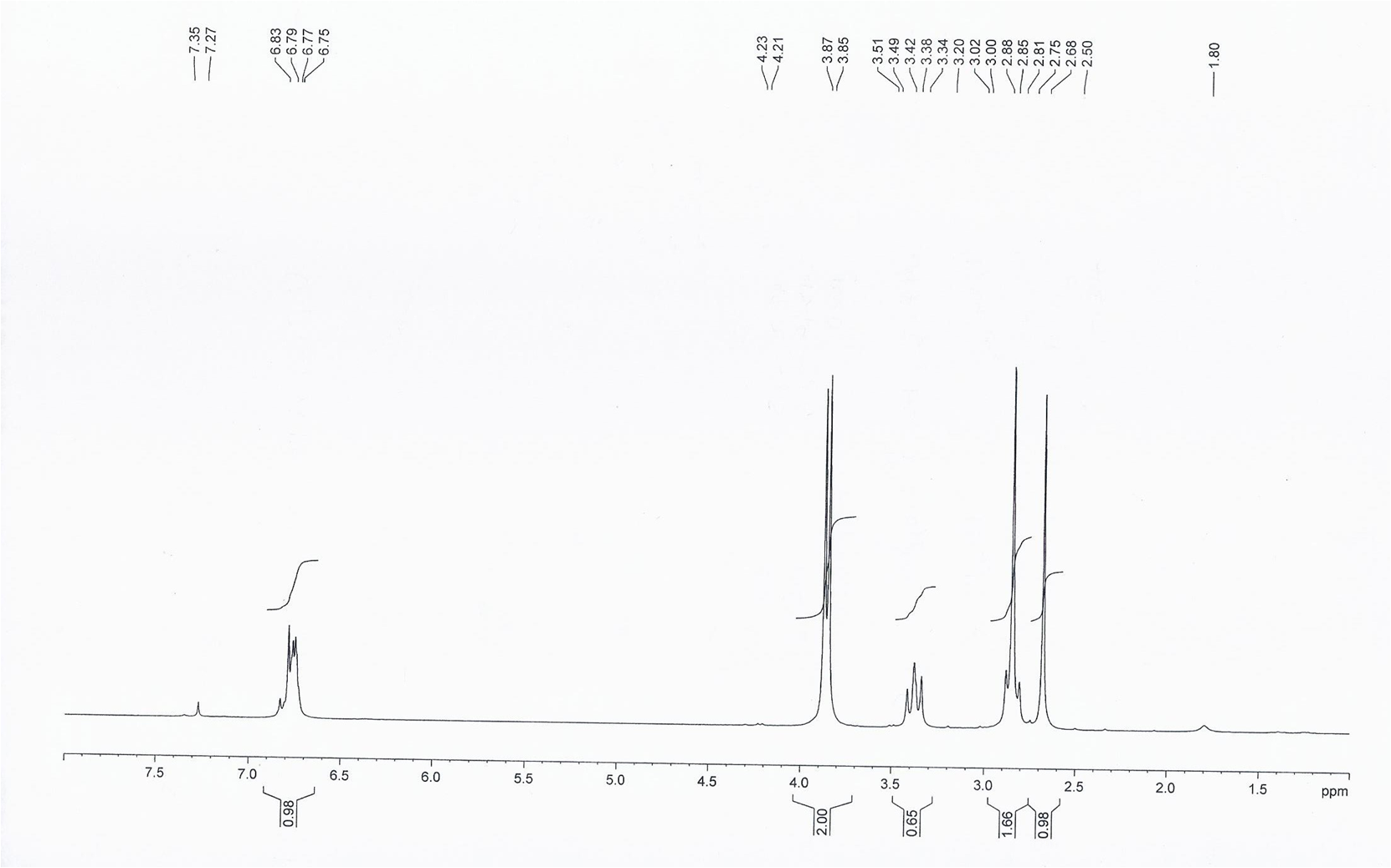

Supplement: S23 Fig — (TIF) [file pone.0162895.s023.tif]
